# Supplementary material for: NDR Functions as a Physiological YAP1 Kinase in the Intestinal Epithelium
Source: Curr Biol. 2015 Feb 2;25(3):296–305. doi: 10.1016/j.cub.2014.11.054 (PMC4426889; doi:10.1016/j.cub.2014.11.054)
Supplement: Document S1. Supplemental Experimental Procedures, Figures S1–S4, and Tables S1–S3 [file mmc1.pdf]

Current Biology, Volume *25*

Supplemental Information

NDR Functions as a Physiological YAP1 Kinase in the Intestinal Epithelium

Lei Zhang, Fengyuan Tang, Luigi Terracciano, Debby Hynx, Reto Kohler, Sandrine Bichet, Daniel Hess, Peter Cron, Brian A. Hemmings, Alexander Hergovich, and Debora Schmitz-Rohmer

Supplemental Information

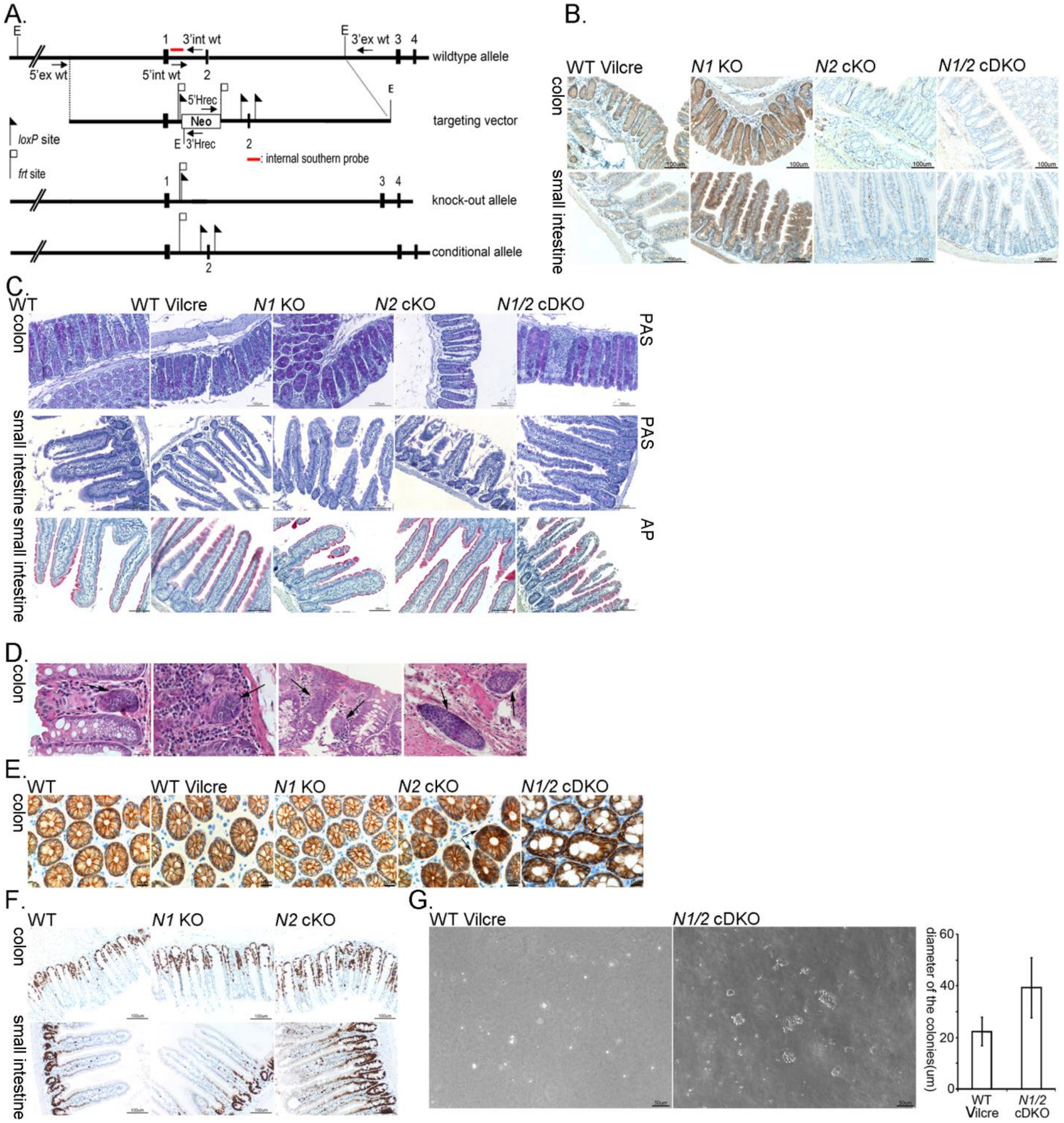

Figure S1 (related to Figure 1). Characterization of the *Ndr* knock-out in the intestinal epithelium

(A) Genomic structure of the *Ndr2* locus in the mouse and targeting vector for conditional *Ndr2* knock-out. Primer binding sites for ES cell screening are indicated (Ex com 5‘/3‘– common external 5‘/3‘ pimer; wt 5‘/3‘ – wild type internal primers; H rec 5‘/3‘ – homologous recombination primers in Neo cassette). E: EcoRI restriction sites used for Southern blot validation of single integration. Binding site of internal southern probe indicated in red. *frt* sites (empty squares) for removal of Neo cassette and *lox*P sites (solid triangles) for removal of Exon 2 are indicated. Note: The generation of the conditional *Ndr2* knock-out mouse line was part of a PhD thesis which can be accessed at http://edoc.unibas.ch/1455/1/Gebundene_Version.pdf.

(B) Validation of intestinal epithelial *Ndr2* knock-out model. NDR2 protein is absent from the intestinal epithelium of *N2* cKO and *N1/2* cDKO mice. Top: colon; bottom: small intestine

(C) Secretory lineage and absorptive enterocytes in *Ndr* single- and double- KO mice are indistinguishable from control mice. Periodic acid Schiff (PAS, top row: colon, middle row: small intestine) and Alkaline Phosphatase (AP, bottom row, small intestine) stainings. Genotypes are indicated above.

(D) Four representative examples of abnormal crypts (indicated by arrows) in *N1/2* cDKO mice present enlarged nuclei, thickened epithelial layers and loss of apical / basal polarity in a subset of cells.

(E) Beta-catenin accumulated crypts (BCAC) in *N2* cKO and *N1/2* cDKO colons, indicated by arrows. Beta-catenin levels in normal crypts are comparable between knock-out and control tissues. Genotypes are indicated above.

(F) Ki67 staining in wild-type and *Ndr* single-knock out intestines. Top: colon; bottom: small intestine. See Fig 1C, D for quantification.

(G) Colony formation assay with primary intestinal epithelial cells freshly isolated from control (WT VilCre) and *N1/2* cDKO colons. Cells from each genotype form similar numbers of colonies (data not shown) but the diameter of *N1/2* cDKO colonies is significantly bigger. n = 50; $p = 7.4 * 10^{-8}$

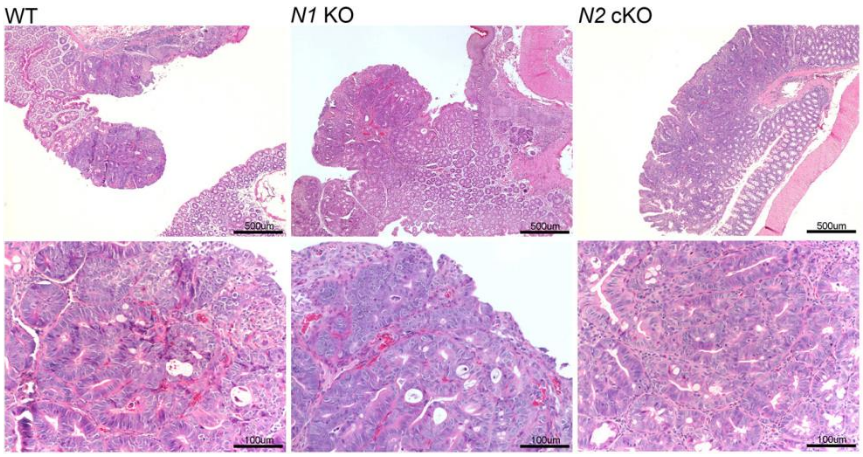

Figure S2 (related to Figure 2). Wild-type and *Ndr* single knock-out mice develop adenocarcinoma upon AOM/DSS treatment.

H&E stained sections of colon nodules in AOM/DSS-treated mice of indicated genotypes after dissection at d64 (see main Figure 2A for treatment protocol). Two different magnifications are shown.

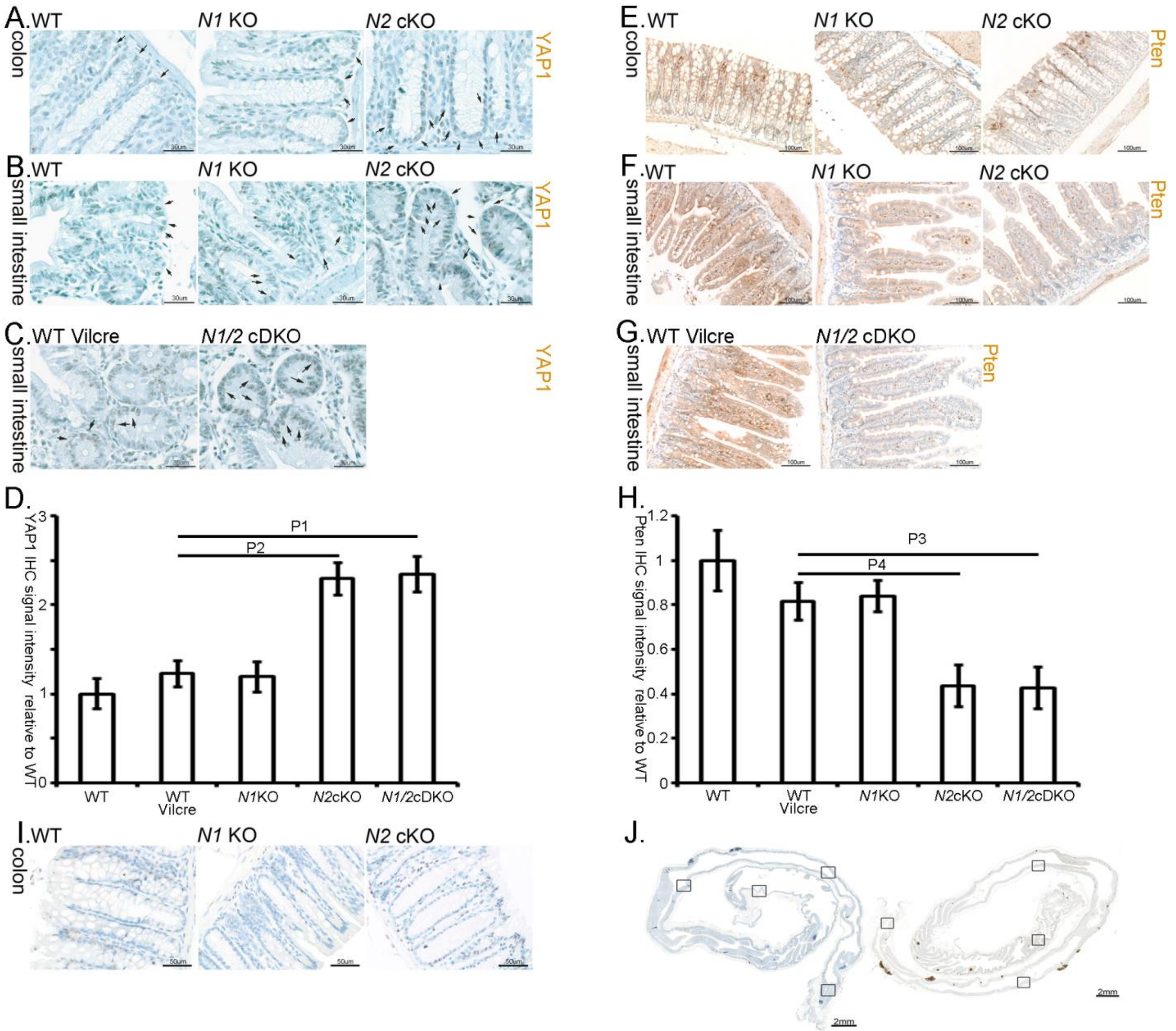

Figure S3 (related to Figure 4). YAP1 and PTEN IHC staining in *Ndr* knock-out mice.

(A), (B), (C) YAP1 protein (brown) detected by IHC in the intestinal epithelium. Genotypes are indicated above, tissue origin on the left.

(D) Quantification of YAP1 IHC signal in the colon (Fig 4B (WT Cre, *N1/2* cDKO) and S3A (WT, *N1* KO, *N2* cKO)). Signal in WT was set as 1. Details of the quantification

method are provided in the Supplemental Experimental Procedures section. T-test: p1 = 2 * 10^{-3}; p2 = 2 * 10^{-3}

(E), (F), (G) Pten protein (brown) detected by IHC in the intestinal epithelium. Genotypes are indicated above, tissue origin on the left.

(H) Quantification of Pten IHC signal in the intestinal epithelium (Fig 4C (WT Cre, *N1/2* cDKO) and S3E - G (WT, *N1* KO, *N2* cKO)). Signal in WT was set as 1. Details of the quantification method are provided in the Supplemental Experimental Procedures section. T-test: p3 = 3 * 10^{-3}; p4 = 3 * 10^{-3}

(I) *Bdnf in situ* hybridization of wild-type and *Ndr* single knock-out colons.

(J) Squares indicate areas taken for quantification of *Bdnf* signal in control (WT VilCre) and *N1/2* cDKO colons – quantification results shown in Fig 4D. Details of the quantification method are provided in the Supplemental Experimental Procedures section.

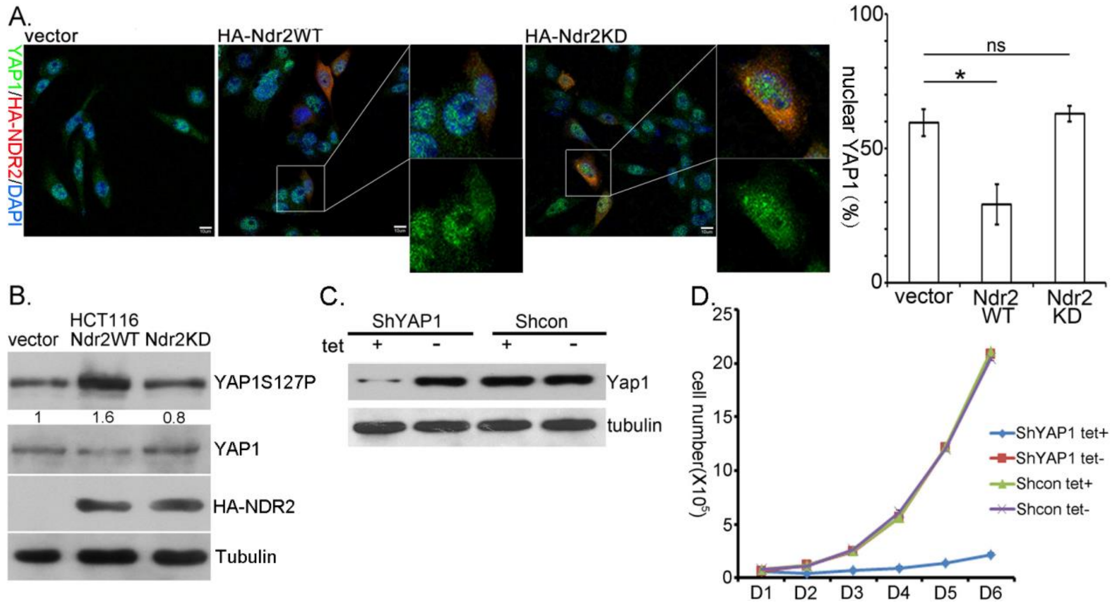

Figure S4 (related to Figure 6). Transient overexpression of active NDR changes YAP regulation

(A) Transient overexpression of wild-type (WT) but not kinase-dead (KD) HA-Ndr2 reduces nuclear localization of endogenous YAP1 in SW480 cells. Quantification on the right; n = 50 cells per condition. p = 0.002.

(B) Overexpression of wild-type (WT) but not kinase-dead (KD) Ndr2 increases S127 phosphorylation of endogenous Yap1 in HCT116 cells.

(C) Validation of tet-induced YAP1 knock-down in SW480 cells.

(D) Tet-induced knock-down of YAP1 reduces proliferation of SW480 cells as reported previously [S1].

Table S1

Mouse ID	*Ndr1*	*Ndr2*	nodule number
1	-/-	Δ/Δ	13
2	-/-	Δ/Δ	18
3	-/-	Δ/Δ	17
4	-/-	Δ/Δ	16
5	-/-	Δ/Δ	15
6	+/+	+/+	2
7	+/+	+/+	0
8	+/+	+/+	2
9	+/+	+/+	6
10	+/+	+/+	1
11	+/+	Δ/Δ	6
12	+/+	Δ/Δ	7
13	+/+	Δ/Δ	5
14	-/-	+/+	3
15	-/-	+/+	1
16	-/-	+/+	2

Number of colonic nodules formed in mice with indicated genotypes upon AOM/DSS treatment. Δ denotes conditional ablation of *Ndr2* in the intestinal epithelium. All mice were female and carried the Vilin-cre transgene.

Table S2

p-site	Peptide Sequence	Mascot Score
S109	QAsTDAGTAGALTPQHVR	60.07
S61 or T63	GD[set]DLEALFNAVMNPK	102.6
S127 or S128 or S131	AH[ssPAs]LQLGAVSPGTLTPT GVVSGPAATPTAQHLR	46
S163 or S164	QsSFEIPDDVPLPAGWEMAK	46.68
	QSsFEIPDDVPLPAGWEMAK	48.89

Identification of additional sites on YAP1 that are phosphorylated by recombinant NDR.

YAP1 was subjected to *in vitro* phosphorylation by NDR, and subsequently purified by SDS-PAGE. Yap1 bands were excised from the gel, subjected to tryptic digest and analyzed by NanoLC-MS/MS on a LTQ Orbitrap Velos (Thermo Scientific) as described [S2]. The phosphorylated peptides were identified with Mascot searching Swiss-Prot 2013_11 [S3] and validated with ScaffoldPTM (Proteome Software).

The sequences of the identified phosphopeptides are shown and the phosphorylated amino acids are marked in red. In cases where the phosphorylation could not be unambiguously assigned to a single serine or threonine in a peptide, the region of phosphorylation is marked in brackets and the possible phosphorylation sites are shown in lower case. All peptides were sequenced multiple times and the highest Mascot Score obtained for each peptide is shown.

Table S3

		YAP1 score	
		Low	High
NDR2 score	Low	51	197
	High	43	34

YAP1 and NDR2 protein expression score in tissue microarray samples. Data are shown as bar graphs in Figure 7C.

Supplemental Experimental Procedures

Histology

Animals were sacrificed, intestines removed, rinsed in cold PBS and fixed overnight in 10% formalin. Following dehydration, tissues were embedded in paraffin in a tissue processor (Medite, TCP15). 3 um sections were cut on a microtome (Microm HM355S) and stained with hematoxylin and eosin (H&E) or used for immunohistochemical stainings.

Alkaline phosphatase activity was assessed with the Vector Red Alkaline Phosphatase Substrate Kit I (Vectorlabs) according to the manufacturer's instructions and counterstained with hematoxylin. For periodic acid-Schiff (PAS) staining, sections were deparaffinized, immersed in Periodic Acid Solution (Sigma) for 5 min at room temperature, washed in water, and incubated in Schiff's reagent (Sigma) for 15 min at room temperature. Sections were washed and counterstained with hematoxylin.

Immunohistochemistry (IHC)

IHC stainings were performed on the Ventana DiscoveryXT instrument (Roche Diagnostics) with either Research IHC DAPMap XT (Ki67, β-catenin, Pten and Ndr2) or Research IHC BlueMap XT (YAP1) procedures. Briefly, for Ndr2 (1:50) and Pten (1:100) slides were pre-treated with standard CC1 (Roche Diagnostics), for β-catenin (1:400) slides were pre-treated with standard CC2 (Roche Diagnostics), for Ki67 (1:50) and Yap1 (1:200) slides were pre-treated with mild CC1 (Roche Diagnostics). Detection of primary antibodies was obtained by manually adding an anti-rabbit polymer conjugated

with HRP (ImmPress Vector) for Ndr2, Pten, β-catenin and Ki67, whereas detection of Yap1 primary antibody was obtained by adding a biotinylated Donkey anti-rabbit secondary antibody (Jackson Laboratories). In addition, for detection of β-catenin we added a rabbit anti-mouse monoclonal antibody (Epitomics) to reduce non-specific binding of the primary antibody. Finally, DAB Map kit (Roche Diagnostics) was used for visualization of antibodies binding to NDR2, Pten, β-catenin and Ki67 and Blue Map kit was used for YAP1.

Quantification of Ki67-positive cells (Fig 1C, D; S1D)

Five images per sample were collected at equal spacing at 10X magnification. Next, 50 crypts were randomly selected for each mouse and Ki67-positive cells were counted with the software Fiji [S4]. The data shown represent the average number of Ki67-positive cells in a given genotype based on five mice analyzed per genotype.

Quantification of YAP and Pten IHC (Fig 4B, C and S3A – H)

Five mice were analyzed per genotype. One section per mouse was photographed and images were segmented into different channels using the Ilastik program (Sommer, C; Straehle C; Koethe U; Hamprecht FA (2011). "ilastik: Interactive Learning and Segmentation Toolkit". *IEEE International Symposium on Biomedical Imaging*: 230–33.). Segmented images where further processed with Fiji [S4] to obtain the percentage of total area covered by YAP or Pten IHC signal. Values obtained for each genotype were normalized to the WT value.

mRNA in situ hybridization with ACD probes and quantification

In Situ Hybridization (ISH) was performed on Ventana Discovery XT instrument using RNAscope technique with the following probes: BDNF (ACD # 316036) and CTGF (ACD # 314546) on 5 um thick colon and small intestine paraffin sections. Briefly, slides were heated to 60°C for 45min and dewaxed up to 100% EtOH offline and air dried. Next, sections were put into pre-heated pretreat2 buffer (ACD) and heated to 98°C for 8 minutes in a microMED T/T Mega (Milestone) microwave. Slides were rinsed in water and put into the instrument for automated staining. The procedure (mRNA DAB DXT 2.0) follows this setting: pre-treatment 2 for 4 min, pre-treatment 3 for 8 min, hybridization with the probes at 48°C for 2h 32 min, Amp5 for 24 min. DapB (ACD # 310048) was used as negative control probe and PPIB (ACD # 313916) was used as positive control probe. BDNF ISH slides were scanned with an Axioscan scanner (Zeiss) and images with an area of $1mm^2$ from cecum (1 image), colon (2 images) and rectum (1 image) were taken for quantification as indicated in Fig S3J. Images were segmented into different channels using the Ilastik program ("ilastik: Interactive Learning and Segmentation Toolkit". *IEEE International Symposium on Biomedical Imaging*: 230–33.). Segmented images where further processed with Fiji [S4] to obtain the ratio of the total tissue area covered by BDNF signal.

Isolation and colony formation of murine intestinal epithelial cells (IEC)

IECs were isolated according to a protocol established by Whitehead et al. [S5]. Mice were sacrificed, colons were removed and placed in PBS. Contents of the colon were removed with PBS using a 10-ml syringe, then washed once with cold PBS containing

0.02% NaClO. Following transfer to a clean dish, colons were washed three times with fresh cold PBS to remove the NaClO. Colons were opened longitudinally and then cut into small pieces with a scalpel. The colonic epithelium was detached using forceful shaking in 0.5 mM EDTA and 0.5 mM dithiothreitol (DTT) in PBS for 30 minutes at 4°C. Muscle tissue fragments were allowed to settle down by gravity for 2 minutes. The supernatant containing the IECs was collected and treated with collagenase (75 ug / ml), protease (20 ug / ul) and Dnase (20 ug /ml) for 15 Minutes at 37°C to generate a single-cell suspension. Next, cells were washed three times with fresh PBS to remove all enzymes. Cells were seeded into agarose plates containing 0.1% agarose in RPMI1640 with 10% FBS. Cells were cultured at 37°C, 5% CO_2, for 10 days to form colonies. An LSM700 confocal system was used to acquire pictures for quantification. Ten images were collected randomly at equal spacing at X20 magnification and colonies were measured and quantified by the IMS image processing software.

Plasmids and antibodies

Antibodies used in the study were obtained from the following suppliers: anti-Ki67 antibody (RM-9106) – Thermo Scientific; anti-YAP1 (#4912), anti-YAP1S127P (#4911), anti-Pten (#9559), anti-LATS1 (# 9153), anti-LATS1S909P (#9157), anti-LATS1T1079P (#8654), anti-MST1 (#3682), anti-MST1/2(T180/T183)P (#3681) – Cell Signaling Technology; anti-β-catenin (14/Beta-Catenin) – BD Transduction Laboratories; anti-YAP1 (sc-15407) for immuno-histochemistry (IHC) – Santa Cruz Biotechnology. Anti-HA Tag antibody (12CA5) – home-made. Rabbit polyclonal anti-NDR1, anti-NDR and anti-p444/2 were generated by us and have been published previously ([S6, S7]).

pcDNA3.0-HA-Ndr1 (wild-type), pcDNA3.0-HA-Ndr1 (kinase dead), pcDNA3.0-HA-Ndr2 (wildtype), pcDNA3.0-HA-Ndr2 (kinase dead) for expression in mammalian cells have been described [S8]. GST-tagged NDR1 PIF (wild-type and kinase-dead, [S9]) were cloned into pFastbac1 and expressed in Sf9 insect cells. pGEX3X-hYAP1 was used for bacterial expression of GST-tagged YAP1. The TEAD-luciferase reporter plasmid was obtained from Addgene (Plasmid 34615; 8xGTIIC-luciferase). pGIPZ-Sh Yap1 and pGIPZ-Sh Luc plasmids were a kind gift from Zhou Dawang.

NDR kinase assays

NDR kinase assays were performed as described in [S8]. Briefly, kinase reactions were carried out in 30 µl buffer containing 20 mM Tris, pH 7.5, 10 mM $MgCl_2$, 1 mM benzamidine, 4 µM leupeptin, 1 µM microcystin, 1 mM DTT, 1 µM cyclic AMP-dependent protein kinase inhibitor peptide, 1 µM ATP, 5 µCi P32 with 500 ng GST-NDR kinase and 500 ng GST-YAP1 protein. Reactions were incubated for 30 minutes at 37°C, stopped by addition of 10 ul 5x Lämmli buffer and boiled for 5 minutes at 95°C prior to resolving them on an SDS-PAGE gel. Proteins were transferred to an immobilon-P PVDF membrane (Millipore), exposed to a phospho-screen and read on a phospho-imager.

Cell culture and transfection

SW480 cells were bought from ATCC (CCL-228) and cultured in RPMI1640 medium with 10% FBS. For transfection, 5 *10^5 (6-well dish) or 2*10^6 (10 cm dish) were plated 24h prior to transfecting with lipofectamine 2000 according to the manufacturer's

instructions. Stable SW480 cell lines expressing Ndr2 wild-type, Ndr2 kinase-dead or luciferase upon tetracycline (tet) administration were generated under selection with Blasticidin (10 ug / ml) and G418 (1 mg / ml).

Immunofluorescence

SW480 cells were seeded in chamber-slides and transfected as described above. 24h after transfection, cells were fixed with 4%PFA for 10min on ice. Next, cells were washed once with PBS for 5 minutes, followed by incubation with 0.5% Triton X-100 in PBS for 15 minutes on ice. After two washes 5-minute washes with PBS, cells were blocked with 1% BSA in PBS at 37°C for 30 minutes. Next, cells were incubated over night at 4°C with primary antibody (anti-Yap1 (1/500) from CST (#4912) and anti-HA (12CA5, homemade, 1/200) in PBS containing 1%BSA. Slides were washed twice with PBS, 5 minutes each time. For detection, cells were incubated with Alexa 647 anti-rabbit (1/1000) and Alexa 488 anti-mouse (1/1000) in PBS for 1h at 37°C. After two 5 – minute washes with PBS, cells were incubated with 5ug / ml DAPI in PBS for 2 minutes at RT. Prolong@Gold medium (Invitrogen) was used to mount slides. An LSM700 confocal system was used to analyze slides. To determine the percentage of nuclear YAP (Fig 6A, S6A), 50 HA-positive cells were collected randomly at equal spacing at 40X magnification. YAP1 signal density for the nucleus (based on DAPI co-localization) and YAP signal density for the entire cell were measured with the IMS image processing software.

TEAD-reporter assay

SW480 cells were seeded in 24-well plates at 100000 cells / well 24h prior to triple-transfection with TEAD-Luciferase reporter (470 ng / well), renilla firefly (10 ng / well) and Ndr2WT (60 ng / well) or Ndr2KD (60 ng / well). 24 hours after transfection, cells were lysed and luciferase activity was assayed using the Dual-Luciferase® Reporter Assay System (Promega, E1910) following the manufacturer's instructions.

Proliferation assays

Stable tet-inducible SW480 cell lines were seeded into 6 well dishes at 5000 cells / well on day 0 in tet-containing medium. Medium was changed every 48h and fresh tet was added. At each time point, cells were detached with 0.5 mM EDTA in PBS and counted with a Vi-CELL analyser (Beckmann Coulter).

References

S1. Zhou, D., Zhang, Y., Wu, H., Barry, E., Yin, Y., Lawrence, E., Dawson, D., Willis, J.E., Markowitz, S.D., Camargo, F.D., et al. (2011). Mst1 and Mst2 protein kinases restrain intestinal stem cell proliferation and colonic tumorigenesis by inhibition of Yes-associated protein (Yap) overabundance. Proceedings of the National Academy of Sciences of the United States of America *108*, E1312-1320.

S2. Grzmil, M., Huber, R.M., Hess, D., Frank, S., Hynx, D., Moncayo, G., Klein, D., Merlo, A., and Hemmings, B.A. (2014). MNK1 pathway activity maintains protein synthesis in rapalog-treated gliomas. J Clin Invest *124*, 742-754.

S3. Perkins, D.N., Pappin, D.J., Creasy, D.M., and Cottrell, J.S. (1999). Probability-based protein identification by searching sequence databases using mass spectrometry data. Electrophoresis *20*, 3551-3567.

S4. Schindelin, J., Arganda-Carreras, I., Frise, E., Kaynig, V., Longair, M., Pietzsch, T., Preibisch, S., Rueden, C., Saalfeld, S., Schmid, B., et al. (2012). Fiji: an open-source platform for biological-image analysis. Nat Methods *9*, 676-682.

S5. Whitehead, R.H., Demmler, K., Rockman, S.P., and Watson, N.K. (1999). Clonogenic growth of epithelial cells from normal colonic mucosa from both mice and humans. Gastroenterology *117*, 858-865.

S6. Cornils, H., Stegert, M.R., Hergovich, A., Hynx, D., Schmitz, D., Dirnhofer, S., and Hemmings, B.A. (2010). Ablation of the kinase NDR1 predisposes mice to the development of T cell lymphoma. Science signaling *3*, ra47.

S7. Tamaskovic, R., Bichsel, S.J., Rogniaux, H., Stegert, M.R., and Hemmings, B.A. (2003). Mechanism of Ca2+-mediated regulation of NDR protein kinase through autophosphorylation and phosphorylation by an upstream kinase. J Biol Chem *278*, 6710-6718.

S8. Hergovich, A., Bichsel, S.J., and Hemmings, B.A. (2005). Human NDR kinases are rapidly activated by MOB proteins through recruitment to the plasma membrane and phosphorylation. Mol Cell Biol *25*, 8259-8272.

S9. Stegert, M.R., Tamaskovic, R., Bichsel, S.J., Hergovich, A., and Hemmings, B.A. (2004). Regulation of NDR2 protein kinase by multi-site phosphorylation and the S100B calcium-binding protein. J Biol Chem *279*, 23806-23812.
