# Supplementary material for: NDR Functions as a Physiological YAP1 Kinase in the Intestinal Epithelium
Source: Curr Biol. 2015 Feb 2;25(3):296–305. doi: 10.1016/j.cub.2014.11.054 (PMC4426889; doi:10.1016/j.cub.2014.11.054)
Supplement: Document S2. Article plus Supplemental Information [file mmc2.pdf]

Current Biology

Article

NDR Functions as a Physiological YAP1 Kinase in the Intestinal Epithelium

Graphical Abstract

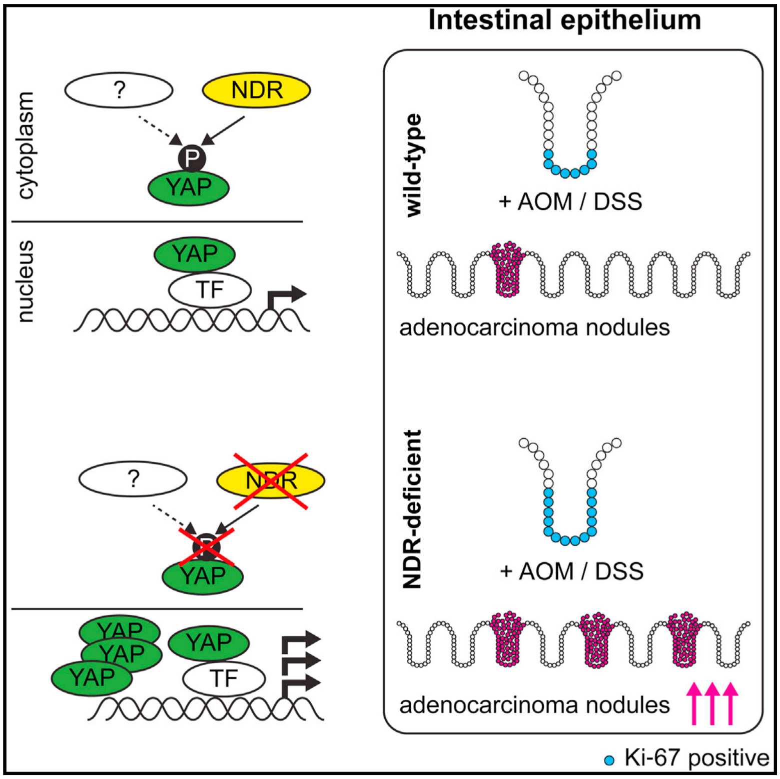

Authors

Lei Zhang, Fengyuan Tang, ...,
Alexander Hergovich,
Debora Schmitz-Rohmer

Correspondence

a.hergovich@ucl.ac.uk (A.H.),
debora.schmitz@fmi.ch (D.S.-R.)

In Brief

Phosphorylation of the Hippo pathway effector YAP1 contributes to tissue homeostasis. However, the identity of the YAP1 kinase in the intestine remains unknown. Here, Zhang et al. report NDR as a physiological YAP1 kinase, restricting YAP1's activity in the intestine and hence establishing the first mouse model of a direct YAP1-S127 kinase.

Highlights

- Mammalian NDR kinases phosphorylate YAP1 on serine 127
- Phosphorylation of YAP1 by NDR kinases regulates YAP1 activity in vivo
- NDR kinases function as tumor suppressors in the intestinal epithelium
- *Ndr* knockout mice represent the first animal model of a direct S127 kinase

Zhang et al., 2015, Current Biology *25*, 296–305
February 2, 2015 ©2015 Elsevier Ltd All rights reserved
http://dx.doi.org/10.1016/j.cub.2014.11.054

Current Biology 25, 296–305, February 2, 2015 ©2015 Elsevier Ltd All rights reserved http://dx.doi.org/10.1016/j.cub.2014.11.054

Article

NDR Functions as a Physiological YAP1 Kinase in the Intestinal Epithelium

Lei Zhang,[1] Fengyuan Tang,[1] Luigi Terracciano,[2] Debby Hynx,[1] Reto Kohler,[3] Sandrine Bichet,[1] Daniel Hess,[1] Peter Cron,[1] Brian A. Hemmings,[1] Alexander Hergovich,[4,*] and Debora Schmitz-Rohmer[1,*]
[1]Friedrich Miescher Institute for Biomedical Research, Maulbeerstrasse 66, 4058 Basel, Switzerland
[2]Institute of Pathology, University of Basel, Schoenbeistrasse 40, 4031 Basel, Switzerland
[3]Department of Biomedicine, University Hospital Basel, Hebelstrasse 20, 4031 Basel, Switzerland
[4]UCL Cancer Institute, University College London, London WC1E 6BT, UK

Summary

Background: Phosphorylation of the transcriptional coactivator YAP1 is a key event in defining Hippo signaling outputs. Previous studies demonstrated that phosphorylation of YAP1 at serine 127 (S127) sequesters YAP1 in the cytoplasm and consequently inhibits YAP1 transcriptional activity. Mammalian tissue-culture experiments suggest that downstream of MST1/2 signaling, LATS1/2 function as YAP1-S127 kinases. However, studies of *Mst1/2* knockout mouse models revealed that the identity of the physiological YAP1-S127 kinase(s) in certain tissues, such as the intestine, remains unknown.
Results: We show that mammalian NDR1/2 kinases phosphorylate YAP1 on S127 and thereby negatively regulate YAP1 activity in tissue-cultured cells. By studying NDR1/2-deficient mice, we demonstrate the in vivo relevance of NDR1/2-mediated regulation of YAP1. Specifically, upon loss of NDR1/2 in the intestinal epithelium, endogenous S127 phosphorylation is decreased whereas total YAP1 levels are increased. Significantly, ablation of NDR1/2 from the intestinal epithelium renders mice exquisitely sensitive to chemically induced colon carcinogenesis. Analysis of human colon cancer samples further revealed that NDR2 and YAP1 protein expression are inversely correlated in the majority of samples with high YAP1 expression. Collectively, we report NDR1/2 as physiological YAP1-S127 kinases that might function as tumor suppressors upstream of YAP1 in human colorectal cancer.
Conclusions: We establish mammalian NDR1/2 as bona fide kinases that target YAP1 on S127 in vitro and in vivo. Our findings therefore have important implications for a broad range of research efforts aimed at decoding and eventually manipulating YAP1 biology in cancer settings, regenerative medicine, and possibly also noncancer human diseases.

Introduction

The transcriptional coactivator YAP1 and its fly counterpart Yorkie drive tissue and organ growth in flies and mammals [1]. Originally delineated in flies, the Hippo kinase phosphorylates the Lats/Warts kinase, which in turn restricts Yorkie activity by phosphorylating serine 168 (S168) [2]. The core of the mammalian Hippo pathway is composed of MST1/2 and LATS1/2 kinases, the mammalian Hippo and Lats/Warts homologs. MST1/2 phosphorylate LATS1/2, which in turn phosphorylate YAP1 on serine 127 (S127), the mammalian equivalent of Yorkie S168 [3], resulting in cytoplasmic retention and decreased transcription of YAP1 target genes [4]. Overexpression studies of YAP1 in transgenic mice revealed YAP1 as a player in cellular transformation in vivo [5, 6]. Subsequent mouse models demonstrated that MST1/2 kinases are required to suppress the oncogenic potential of YAP1 in the liver and the intestinal epithelium [7–10], yet none of these models provided strong evidence for LATS1/2 as direct YAP1-S127 kinases. One study suggested that in the liver, MST1/2 activate a kinase distinct from LATS1/2 to phosphorylate YAP1 on S127 [9]. Another study did not address how deregulation of MST1/2 impacts on S127 phosphorylation in the liver [8]. In the intestinal epithelium, ablation of MST1/2 kinase activity results in YAP1-dependent crypt hyperplasia [10, 11]. However, the YAP1-S127 kinase functioning downstream of MST1/2 was not addressed in the intestinal epithelium [10, 11].

Mammalian NDR kinases are the closest homologs of LATS1/2 [12]. The mammalian genome encodes two highly similar isoforms, NDR1 and NDR2, which are located at distinct genomic loci [13]. Functionally, NDR kinases have been reported to regulate centrosome duplication [14, 15], apoptosis [16, 17], proliferation [18], and chromosome alignment [19, 20] in tissue-cultured cells. We previously published that loss of *Ndr1* predisposes mice to T cell lymphoma development [16], suggesting a tumor-suppressive function of mammalian NDR kinases. The same study showed that NDR2 protein, but not mRNA, is upregulated upon genetic *Ndr1* deletion, suggesting that compensatory effects between the two mammalian NDR isoforms prevent the discovery of additional physiological functions of NDR kinases.

In summary, in the intestinal epithelium, direct physiological regulators of YAP1 by phosphorylation are currently unknown. The present study shows that mammalian NDR kinases phosphorylate YAP1 on S127 in vitro in tissue-cultured cells and in vivo in the intestinal epithelium. Phosphorylation of YAP1 at S127 by NDR mediates sequestration of YAP1 in the cytoplasm, restricts the transcriptional coactivator function of YAP1, and suppresses proliferation of human colon cancer cells.

Results

Mammalian NDR Kinases Restrain Proliferation and Hyperplastic Growth in the Intestinal Epithelium

NDR1 protects against T cell lymphoma development and is highly expressed in organs of the immune system. Conversely, NDR2 is most abundant in the gastrointestinal tract [13, 16]. Therefore, we hypothesized that NDR2 might function as a tumor suppressor in the colonic epithelium. To test this hypothesis and avoid compensation by NDR1, we ablated NDR2 specifically in the intestinal epithelium of *Ndr1* null mice (Figure S1A available online) [16, 21]. Specific ablation of NDR2 in the intestinal epithelium, denoted *N2* cKO (conditional

*Correspondence: a.hergovich@ucl.ac.uk (A.H.), debora.schmitz@fmi.ch (D.S.-R.)

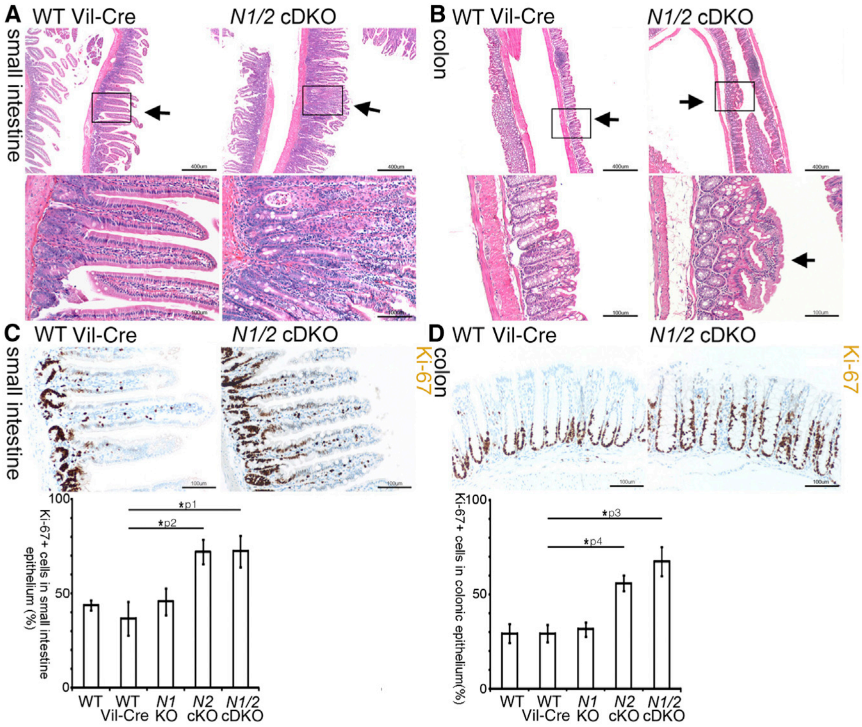

Figure 1. Mammalian NDR Kinases Restrain Proliferation and Hyperplastic Growth in the Intestinal Epithelium

(A and B) Hyperplastic patches in the intestinal epithelium of *N1/2* cDKO (*Ndr1*$^{-/-}$*Ndr2*$^{f/f}$ Vil-Cre^{+}) mice (A: small intestine; B: colon) visualized by H&E staining. Larger magnification of the hyperplastic areas and control regions are shown below. Scale bars represent 400 μm (upper panels) and 100 μm (lower panels).

(C and D) Ki-67 IHC staining (brown) in *N1/2* cDKO and control mice (top) and corresponding quantification (bottom) (C: small intestine; D: colon). Numbers represent the average of a total of 200 crypts counted in five mice per genotype. Student's t test: *p1 = 3×10^{-7}, *p2 = 4×10^{-8}, *p3 = 9×10^{-3}, *p4 = 3×10^{-3}. Scale bars represent 100 μm.

See also Figure S1.

single knockout), was confirmed by immunohistochemistry with an NDR2-specific antibody (Figure S1B). *Ndr1* null mice lacking NDR2 in the intestinal epithelium, denoted *N1/2* cDKO (conditional double knockout), were born in the expected Mendelian ratios, developed no obvious morphological abnormalities, and did not display spontaneous tumor formation in the colon (monitored for up to 24 months; data not shown). However, the intestinal epithelium of *N1/2* cDKO adult mice (aged 16–20 weeks) displayed hyperplastic areas, which were absent in *Ndr1* and *Ndr2* single KO and wild-type controls (Figures 1A and 1B; data not shown). Subsequent histological analyses revealed that the secretory lineage and absorptive enterocytes in *N1/2* cDKO mice were indistinguishable from control animals (Figure S1C). Taken together, these findings suggest that mammalian NDR prevents hyperplastic growth in the intestinal epithelium but is dispensable for differentiation of the secretory lineage and absorptive enterocytes.

Further histological analyses of *N1/2* cDKO intestines revealed the presence of aberrant crypts, characterized by enlarged nuclei, a thickened epithelial cell layer, and loss of apical-basal polarity (Figure S1D). These abnormal crypts resembled so-called β-catenin-accumulated crypts (BCACs), which have been proposed as biomarkers for colon carcinogenesis in rodents and humans [22]. Indeed, we detected increased β-catenin levels in aberrant crypts in *N2* cKO and *N1/2* cDKO but not in wild-type, wild-type-Vil-Cre, or *N1* KO mice (Figure S1E), suggesting that NDR2 ablation can result in BCAC formation.

To characterize the hyperplastic phenotype of *N1/2* cDKO intestines further, we determined the proliferation index of *N1/2* null and control epithelia (Figures 1C and 1D; Figure S1F). The entire proliferative zone of *N1/2* null colonic epithelia in both normal and hyperplastic areas was extended 2-fold compared to wild-type and *Ndr1* single KO mice. *N2* cKO colonic epithelia displayed an intermediate increase in proliferation, indicating that NDR1 compensates only partially for NDR2 loss in this compartment. To further analyze the proliferative behavior of NDR-deficient intestinal epithelial cells (IECs), we performed colony formation assays as described [23] revealing that primary IECs isolated from *N1/2* cDKO mice formed similar numbers of colonies as controls (data not shown). However, *N1/2* null colonies grew significantly larger than wild-type colonies (Figure S1F). In summary, our data indicate that mammalian NDR kinases restrict proliferation of intestinal epithelial cells in vivo.

NDR Protects Mice against Azoxymethane/Dextran Sodium Sulfate-Induced Colon Carcinogenesis

Although *N1/2* cDKO mice developed intestinal hyperplasia and BCACs (Figure 1; Figures S1C and S1D), we did not observe progression to adenocarcinoma in the genetic background analyzed (mixed C57B/L6-Ola129). Spontaneous colon cancer development in rodents is rare [24]. Therefore, we assessed whether NDR protects mice against chemically induced colon carcinogenesis [25]. *Ndr* KO and control mice were treated with the colonotropic mutagen azoxymethane (AOM) and the inflammatory agent dextran sodium sulfate (DSS) as depicted in Figure 2A. Regardless of NDR status, all but one control mouse developed colonic nodules, indicating that the treatment reproducibly induced colon carcinogenesis. A representative pair of AOM/DSS-treated *N1/2* cDKO versus control colon is shown in Figure 2B. Based on histopathological analysis, all nodules analyzed were diagnosed as adenocarcinoma (Figure 2C; Figure S2). However, whereas control mice (WT Vil-Cre) developed 2 or 3 nodules throughout their colon, *N1/2* cDKO mice developed 16 nodules on average (Figure 2D; Table S1). Although nodule lumping precluded rigorous quantification of nodule size in *N1/2* cDKO epithelia, isolated nodules appeared to be of similar size in KO and control animals at dissection (data not shown). Ablation of *Ndr2* alone increased the average nodule number per mouse to six, whereas *Ndr1* single KO had no effect on nodule numbers compared to controls (Figure 2D). Intriguingly, these observations fully parallel our in vivo proliferation measurements (Figures 1C and 1D), namely that loss of NDR2 alone, but not loss of NDR1, increases proliferation in the colonic epithelium, which is further increased in *N1/2* cDKO mice (Figure 1D).

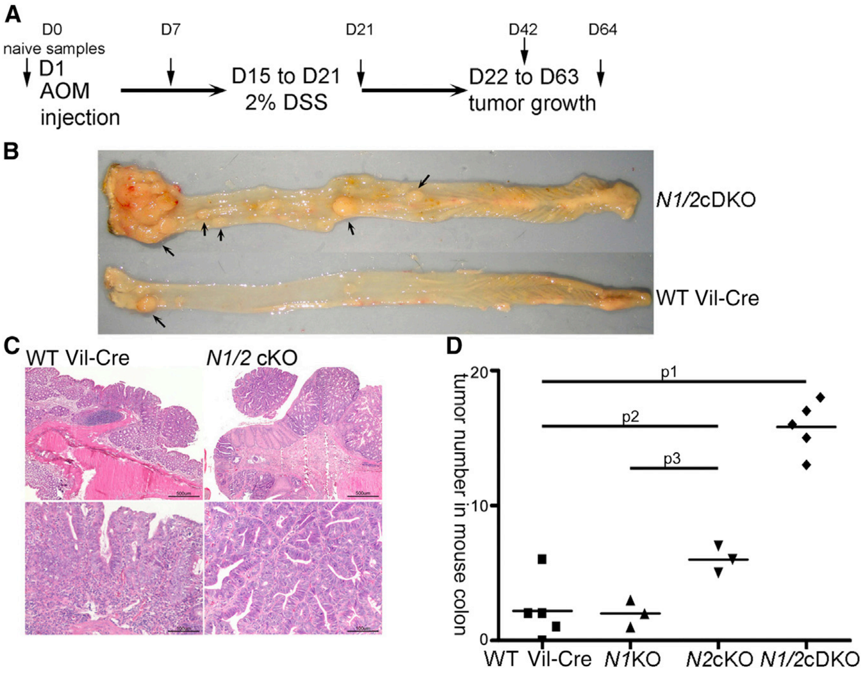

Figure 2. NDR Kinases Protect against AOM/DSS-Induced Colon Carcinogenesis

(A) AOM/DSS treatment protocol. Arrows indicate time points of tissue analysis in wild-type mice (see Figure 3). D, day.

(B) Representative pair of *N1/2* cDKO and control colons post-AOM/DSS treatment after dissection at day 64.

(C) H&E-stained sections of colon nodules in AOM/DSS-treated *N1/2* cDKO and control mice after dissection at day 64. Scale bars represent 500 μm (upper panels) and 100 μm (lower panels).

(D) Quantification of colon nodule numbers in *N1/2* cDKO and control mice post-AOM/DSS treatment after dissection at day 64. Lines indicate the average tumor number in a given group. Student's t test: $p1 = 9 \times 10^{-6}$, $p2 = 0.02$, $p3 = 8 \times 10^{-3}$.

See also Figure S2 and Table S1.

Taken together, our data indicate that mammalian NDR kinases suppress tumor initiation in the intestinal epithelium.

NDR2 Protein Is Progressively Lost during AOM/DSS-Induced Colon Carcinogenesis

Having shown that NDR partially protects against AOM/DSS-induced carcinogenesis in *Ndr* KO mice, we next took the reverse approach and applied the AOM/DSS model to wild-type mice to assess whether NDR2 expression was lost during colon carcinogenesis. We subjected wild-type mice to the AOM/DSS protocol described in Figure 2A. Changes in the colonic epithelium were monitored at the time points indicated: prior to the AOM injection (day 0), day 7, day 21 (upon completion of DSS administration), day 42, and at termination (day 64). AOM treatment induces changes in β-catenin localization [26, 27], a hallmark of human colorectal cancer [28]. Therefore, we assessed β-catenin expression together with hematoxylin and eosin (H&E) and Ki-67 staining to evaluate molecular, morphological, and proliferative changes. In parallel, we monitored NDR2 protein expression using our isoform-specific antibody (Figure S1B). As expected, naive control mice displayed an intact colonic epithelium and cytoplasmic β-catenin localization and proliferating cells were confined to the crypt base (Figure 3A, I, III, and IV). NDR2 expression was detected throughout the entire epithelium (Figure 3A, II). One week after AOM injection (Figure 3B), tissue architecture, β-catenin localization, and NDR2 levels remained unchanged whereas the Ki-67-positive compartment appeared slightly extended upward (Figure 3B, IV). After DSS treatment (day 21), the colonic epithelium was destroyed and crypt regeneration was apparent (Figure 3C). Some crypts showed aberrant morphology (Figure 3C, I). β-catenin signals were increased and occasionally nuclear (Figure 3C, III). Aberrant crypts displayed strong Ki-67 expression, indicating elevated proliferation rates (Figure 3C, III and IV). NDR2 levels appeared generally decreased, in line with our hypothesis that NDR restricts proliferation in the intestinal epithelium (Figure 3C, II). On day 42, aberrant crypts had formed in the regenerated colonic epithelium (Figure 3D, I). We did not detect adenoma or adenocarcinoma at this time point. β-catenin and Ki-67 signals were comparable to the previous time point (day 21) (Figure 3D, III and IV). However, NDR2 levels in aberrant crypts were diminished compared to adjacent normal crypts (Figure 3D, II). Nine weeks after AOM injection (day 64), adenocarcinomas had formed and β-catenin signals were strongly increased, displaying nuclear localization (Figure 3E, I and III). The majority of cells within the nodules were Ki-67 positive (Figure 3E, IV). Whereas stroma cells retained residual NDR2 levels, NDR2 protein was absent in the majority of adenocarcinoma nodules (Figure 3E, II). Importantly, NDR2 protein levels in adjacent normal crypts were comparable to those detected prior to AOM treatment (compare Figure 3A, II and Figure 3E, II). In summary, NDR2 protein is progressively lost during AOM-induced colon carcinogenesis and absent in adenocarcinoma, supporting our hypothesis that NDR2 functions as a tumor suppressor protein in the colon.

Levels of the YAP1 Oncoprotein Are Increased upon NDR Ablation

The Hippo-YAP1 pathway regulates organ growth [29, 30]. Current evidence suggests that the Hippo core kinase cassette—MST1/2 and LATS1/2 in mammals—inactivates the transcriptional coactivator YAP1 by LATS1/2-mediated phosphorylation. Upon phosphorylation at serine 127 by LATS kinases [4, 6], YAP1 can be retained in the cytoplasm. Phosphorylation of serine 381 (S381) by LATS can trigger YAP1 degradation [31]. In the absence of Hippo pathway activity, YAP1 can enter the nucleus and drive proproliferative gene expression. Although YAP1 is dispensable for normal intestinal development and homeostasis [11], its oncogenic potential is unleashed in the absence of Hippo pathway activity [10, 11]. Importantly, whether LATS1/2 can directly regulate YAP1 in the intestinal epithelium remains to be addressed [10]. NDR kinases are the closest homologs of LATS kinases [12]. Hao et al. [32] reported that human NDR can phosphorylate YAP1 in vitro. However, neither the phosphorylation site(s) nor the biological significance of this phosphorylation event was examined. Therefore, we hypothesized that NDR kinases might regulate YAP1 in the intestinal epithelium, which could explain why *N1/2* cDKO animals are more susceptible to

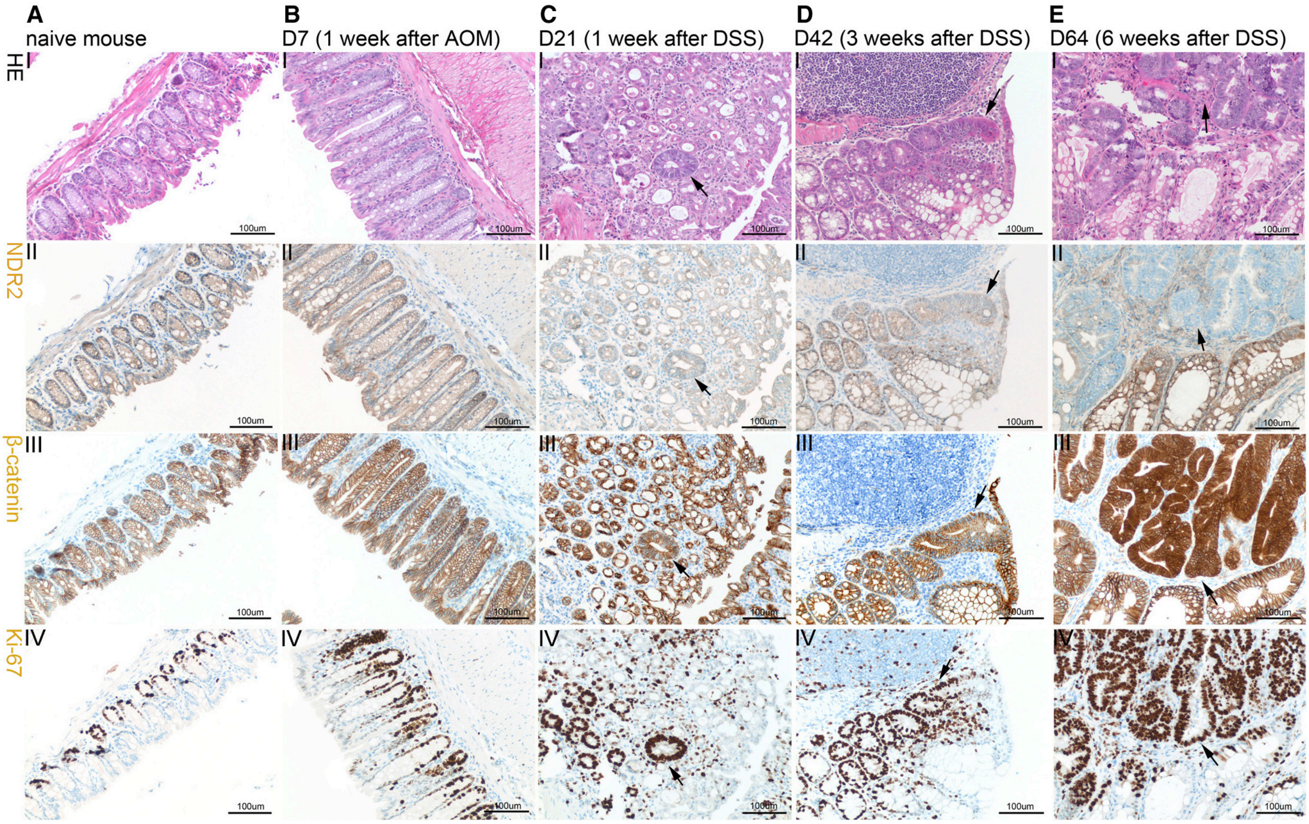

Figure 3. NDR2 Protein Is Progressively Lost during Colon Carcinogenesis

Tissue architecture (H&E) and NDR2, β-catenin, and Ki-67 IHC staining of the colonic epithelium of wild-type C57BL6 mice after AOM/DSS. Serial sections were taken for the different stainings at each time point. Arrows indicate aberrant crypts. Scale bars represent 100 μm. Three mice were analyzed for each time point, as follows.

(A) Prior to AOM injection.

(B) D7: 1 week after AOM intraperitoneal injection.

(C) D21: 1 week after completion of 2% DSS treatment.

(D) D42: 3 weeks after completion of 2% DSS treatment.

(E) D64: 6 weeks after completion of 2% DSS treatment and the end of the time course.

chemically induced tumorigenesis. To examine this hypothesis, we asked whether loss of NDR deregulates YAP1 activity by altering YAP1 phosphorylation in the intestinal epithelium. Murine S112 is the equivalent of human S127 [3]. Significantly, phospho-S112 levels were decreased whereas total YAP1 protein levels were increased in lysates of the intestinal epithelium derived from *N2* cKO and *N1/2* cDKO mice (Figures 4A and 4B). In contrast, phosphorylation of YAP1 at S382, the murine equivalent of human S381, was unaffected (Figure 4A). Importantly, LATS1 total protein levels and LATS activity as determined by phosphorylation status were unchanged. Total MST1 and phospho-MST1/2 (Thr183/180) levels, indicative of MST1/2 activity, also remained unchanged (Figure 4A). These findings demonstrate that the observed decrease of YAP1 phosphorylation was caused by the absence of NDR kinases and not altered MST-LATS signaling. Immunohistochemistry (IHC) staining of tissue sections confirmed the upregulation of YAP1 protein in the intestinal epithelium of *N1/2* cDKO and *N2* cKO mice and revealed that YAP1 is mainly nuclear both in KO and control animals (Figure 4C; Figures S3A–S3D).

To define whether decreased YAP1 phosphorylation and elevated YAP1 protein levels upon loss of NDR translated into increased YAP1 activity in vivo, we employed two complementary approaches. YAP1 overexpression in the intestine correlates with downregulation of the tumor suppressor PTEN [33], exerting its tumor-suppressive functions in part by decreasing Cyclin D1 levels [34]. In agreement with increased YAP1 activity upon NDR loss, we found decreased PTEN and increased Cyclin D1 protein levels in *N1/2* null colons (Figures 4A and 4D; Figures S3E–S3H). As a second approach, we assessed the expression of YAP1 target gene expression in the intestinal epithelium of *N1/2* cDKO mice by in situ hybridization (ISH). CTGF, an established transcriptional target of YAP1/TEAD [35], was only detectable in the intestinal stroma (data not shown), a tissue where *Ndr2* is not deleted by the Villin-Cre transgene in our KO mice. Thus, we examined BDNF, another YAP1 target gene [35], by ISH, revealing that *Bdnf* transcripts were more than 2-fold upregulated in *N1/2* cDKO mice compared to controls (Figure 4E; Figures S3I and S3J), indicating that NDR restrains YAP1 target gene expression in vivo. Taken together, our data suggest that elevated YAP1 levels upon loss of NDR translate into increased YAP1 activity in vivo.

Next, to test the relevance of YAP1 regulation by NDR in the intestinal epithelium, we asked whether the observed hypersensitivity to chemical carcinogenesis of *N1/2* cDKO mice

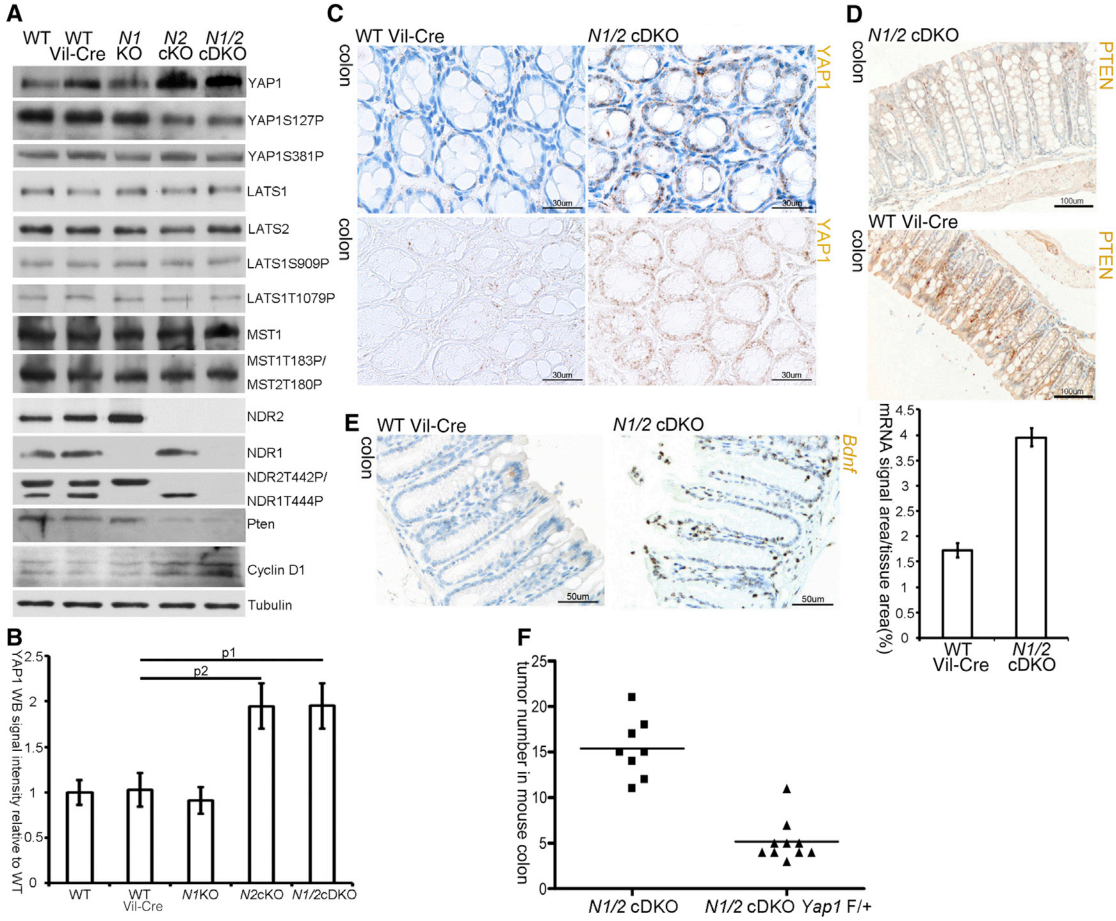

Figure 4. Loss of NDR Causes YAP1 Upregulation in the Intestinal Epithelium

(A) Western blot analysis of total colon lysates using the indicated antibodies.

(B) Quantification of total YAP1 levels from (A) normalized to tubulin in different genotypes relative to WT. Each bar represents the western blot (WB) signal intensity of three different mice. Student's t test: p1 = 0.02, p2 = 0.03.

(C) YAP1 IHC staining (brown) in the colon of *N1/2* cDKO and control mice (WT Vil-Cre). Bottom: YAP1 staining without counterstain. Scale bars represent 30 μm. See Figure S3D for quantification.

(D) PTEN IHC staining (brown) in the colon of *N1/2* cDKO and control mice. Scale bars represent 100 μm. See Figure S3H for quantification.

(E) *Bdnf* in situ hybridization (brown) in the colon of *N1/2* cDKO and control mice and quantification. Student's t test: $p = 3 \times 10^{-4}$. Scale bars represent 50 μm. See also Figures S3I and S3J.

(F) Quantification of colon nodule numbers in *N1/2* cDKO and *N1/2* cDKO mice with heterozygous deletion of *Yap1* post-AOM/DSS treatment after dissection at day 64. Lines indicate the average tumor number in a given group. Student's t test: $p = 6.7 \times 10^{-6}$.

See also Figures 2D and S3.

was functionally linked to YAP1 levels. To address this question, we concomitantly ablated *YAP1* in *N1/2* cDKO mice and subjected them to AOM-mediated colon carcinogenesis as outlined in Figure 2B. Significantly, removal of one *YAP1* allele was sufficient to reduce tumor formation in *N1/2* cDKO mice from 15 to 5 nodules on average (compare Figures 2D and 4F). Therefore, NDR is functionally required to restrict the oncogenic potential of YAP1 in the intestinal epithelium.

NDR Functions as a YAP1 Kinase Phosphorylating YAP1 on Serine 127

Given the intriguing inverse correlation between NDR loss, decreased YAP1 phosphorylation, and increased YAP1 activity in the intestine (Figure 4), we investigated whether NDR impacts directly on YAP1 regulation. To address whether and where NDR phosphorylates YAP1 directly, we performed in vitro kinase assays with recombinant human YAP1 and NDR-PIF, a constitutively active form of NDR [36]. Indeed, active, but not kinase-dead, NDR-PIF phosphorylated YAP1 as determined by autoradiography (Figure 5A, middle panel). Subsequent western blotting revealed that NDR phosphorylates YAP1 on S127 (Figure 5A, top panel), identifying for the first time a YAP1-S127 kinase distinct from LATS1/2. Next, we performed mass spectrometry to determine additional sites on YAP1 targeted by NDR, identifying three additional serines, namely S61, S109, and S164, to also be phosphorylated by NDR in vitro (Table S2). Significantly, all four sites—S61/S109/S127/S164—are also phosphorylated by LATS [4, 32], suggesting that LATS and NDR kinases can function as YAP1 kinases. Collectively, our kinase assays clearly establish NDR kinases as novel bona fide upstream kinases of YAP1 in vitro.

To investigate whether NDR kinases function as YAP1 kinases in mammalian cells, we overexpressed NDR in the colon

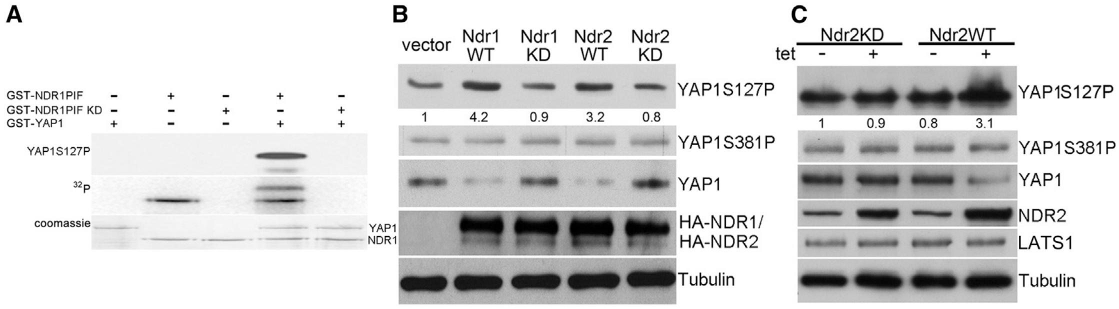

Figure 5. NDR Kinases Phosphorylate hYAP1 on Ser127

(A) Constitutively active NDR1 (glutathione S-transferase [GST]-NDR1-PIF) phosphorylates GST-YAP1 on serine 127. Kinase assays were carried out with the indicated proteins in the presence of radiolabeled ATP (^{32}P). Kinase-dead (KD) NDR1 serves as negative control. Top: western blot with phospho-S127 YAP1 antibody; middle: autoradiography; bottom: Coomassie (loading control). See also Table S2.

(B) Endogenous protein levels of phospho-S127 YAP1, phospho-S381 YAP1, and total YAP1 in SW480 cells transiently overexpressing the indicated hemagglutinin (HA)-tagged NDR constructs. Phospho-S127 YAP1 levels (normalized to total YAP1 and vector control) are shown.

(C) Endogenous protein levels of phospho-127 YAP1, phospho-381 YAP1, and total YAP1 in stable SW480 cell pools that overexpress untagged wild-type or kinase-dead NDR2 in a tetracycline-inducible manner. LATS1 levels remained stable. Phospho-S127 YAP1 levels (normalized to total YAP1 and vector control) are shown.

cancer cell line SW480 and assessed S127 phosphorylation (Figure 5B). In line with our hypothesis that NDR regulates YAP1 by phosphorylation, we found that wild-type—but not inactive—NDR increased the ratio of S127 phosphorylation significantly (Figure 5B). Contrarily, S381 phosphorylation was unaffected (Figure 5B). Inducible overexpression of NDR in stably transfected SW480 cells (tet-on system) and transient transfection experiments in HCT116 cells yielded comparable results (Figure 5C; Figure S4B), excluding the possibility that the observed effects are cell line specific. In summary, our data demonstrate that NDR kinases phosphorylate YAP1 directly on serine 127 in vitro and in tissue-cultured cells. Significantly, these findings fully support our initial observation of decreased S127 phosphorylation in *N1/2* cDKO animals in vivo (Figure 4A).

NDR Regulates the Localization and Transcriptional Activity of YAP1

Considering that phosphorylation of YAP1 at S127 can result in the inactivation of YAP1 by cytoplasmic retention [4, 6] and that NDR phosphorylates YAP1 at S127 (Figure 5), we reasoned that NDR overexpression might reduce nuclear YAP1 levels and consequently YAP1 activity as a transcriptional coactivator. To test this hypothesis, we quantified endogenous YAP1 localization by immunofluorescence in our tetracycline (tet)-inducible colon cancer cell lines described above (Figure 5C). The ratio of nuclear versus cytoplasmic YAP1 was significantly reduced in cells overexpressing wild-type NDR compared to controls that overexpressed kinase-dead NDR (Figure 6A). Signals for total YAP1 levels were also reduced upon overexpression of active NDR (Figure 6A). The same was observed in a transient overexpression setting (Figure S4A). Because the exclusion of YAP1 from the nucleus was dependent on NDR kinase activity (Figure 6A; Figure S4A), these results suggest that this regulatory event is controlled by NDR-mediated YAP1 phosphorylation.

Nuclear YAP1 associates with TEAD transcription factors (TFs) to drive target gene expression [1]. Therefore, we assessed whether NDR-mediated YAP1 phosphorylation interferes with endogenous YAP1 transcriptional activity. Making use of a published TEAD luciferase reporter assay [37], we found that overexpression of wild-type NDR led to a 7-fold drop in reporter activity (Figure 6B). Conversely, overexpression of kinase-dead NDR resulted in a 2.7-fold increase, suggesting that catalytically inactive NDR acts in a dominant-negative manner as already reported in another setting [15]. Collectively, these data indicate that NDR can suppress YAP1 activity in a kinase activity-dependent manner, resulting in cytoplasmic retention of YAP1 when NDR kinase is overexpressed.

Considering that YAP1 drives proliferation in colon cancer [10, 38] and that NDR negatively regulates YAP1 levels and activity, we asked next whether NDR negatively affects YAP1-dependent proliferation of human colon cancer cells. In full agreement with a previous report [10], knockdown of YAP1 in SW480 cells reduced proliferation (Figures S4C and S4D), illustrating that proliferation of SW480 cells is YAP1 dependent. Significantly, we observed a similar effect upon tet-induced overexpression of wild-type NDR in SW480 cells (Figure 6C). Overexpression of kinase-dead NDR had no effect (Figure 6C), showing that proliferation was negatively affected in a manner dependent on NDR kinase activity. Moreover, overexpression of wild-type NDR significantly suppressed colony formation in contrast to controls (Figure 6D). Overexpression of kinase-dead NDR had no suppressive effect (Figure 6D). Collectively, these findings demonstrate the negative impact of NDR kinase activity on the proliferative capacity of colon cancer cells. This observation parallels our findings that NDR loss in vivo increases proliferation (Figure 1) and sensitizes mice to adenocarcinoma nodule formation upon exposure to AOM (Figure 2). Mechanistically, these findings demonstrate that forced expression of active NDR results in a redistribution of YAP1 protein to the cytoplasm and decreased transcriptional activity of YAP1. Functionally, our data indicate that active NDR restricts proliferation of colon cancer cells.

Finally, we turned to clinical colon cancer samples to address the relevance of our findings in human patients. Initially, we detected an inverse correlation of NDR2 and YAP1 protein expression in six out of ten adenocarcinoma samples. More specifically, YAP1 expression was elevated in the tumor and low in the adjacent normal crypts, whereas the opposite was true for NDR2 (Figure 7A). To increase the spectrum of our analysis, we assessed YAP1 and NDR2

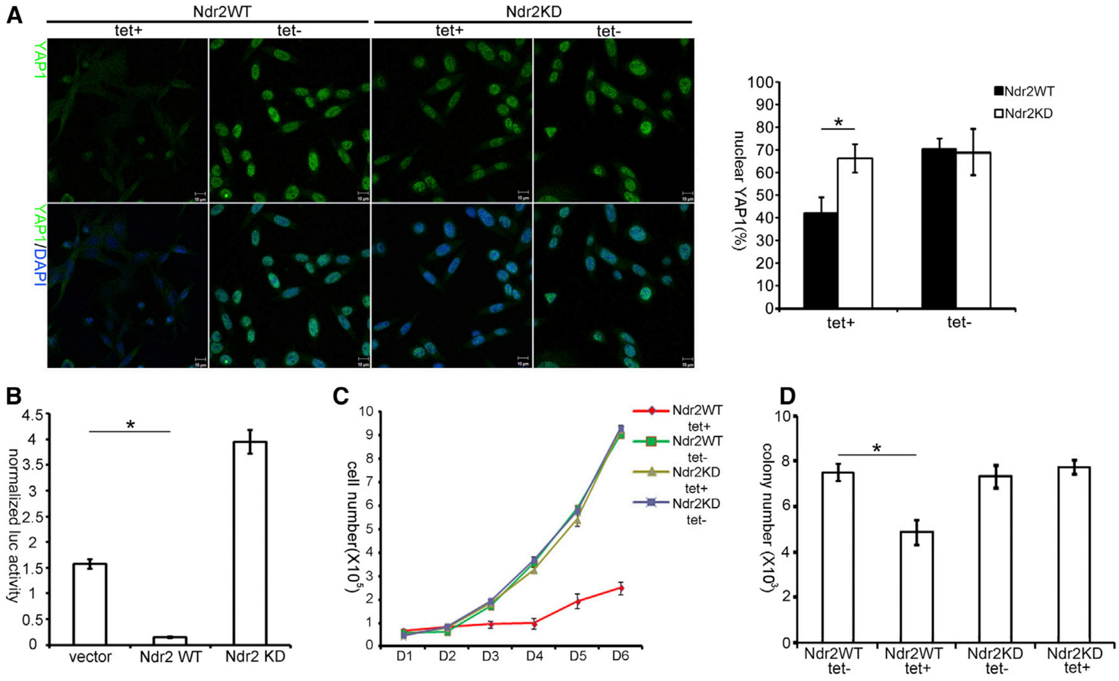

Figure 6. NDR Regulates the Cellular Distribution and Transcriptional Activity of YAP1

(A) Left: cellular distribution of endogenous YAP1 in SW480 cells overexpressing wild-type or kinase-dead Ndr2 in a tet-inducible manner (top: immunofluorescent [IF] staining for YAP1; bottom: merge of IF YAP1 and 4′,6-diamidino-2-phenylindole [DAPI]). Right: quantification of the nuclear/cytoplasmic YAP1 ratio per cell (n = 50 per condition). Student's t test: $*p = 6 \times 10^{-6}$. Scale bars represent 10 μm.

(B) TEAD-luciferase reporter assay in SW480 cells transiently transfected with empty vector, WT, or KD Ndr2. Firefly luciferase activity was normalized to Renilla signal (n = 3). Student's t test: $*p = 1 \times 10^{-7}$.

(C) Proliferation curves of SW480 cells expressing WT or KD Ndr2 in a tet-inducible manner.

(D) Colony formation assay with the same cell lines as in (C). Colony numbers were scored 10 days after seeding (n = 3). Student's t test: $*p = 9 \times 10^{-3}$. See also Figure S4.

protein levels in a tissue microarray with 325 independent human colon cancer samples. Examples of high and low NDR2 and YAP1 scores as determined by IHC are shown in Figure 7B. Significantly, the majority of human tumors with increased YAP1 levels expressed only low amounts of NDR2 whereas NDR2 expression in YAP1-low tumors was variable (Figure 7C; Table S3), in line with our observation that, in mice, YAP1 levels in the intestinal epithelium are increased in the absence of NDR (Figure 4). Collectively, this analysis of clinical samples indicates that NDR2 kinase might also play a tumor-suppressive role in the human intestine.

Discussion

In the present study, we identify mammalian NDR1/2 as novel YAP1 kinases in vitro and in vivo. Recombinant NDR phosphorylates YAP1 on S127 and other reported LATS sites. In tissue-cultured cells, NDR regulates YAP1 function and phosphorylation in a kinase activity-dependent manner. Loss of NDR in the murine intestinal epithelium results in decreased YAP1 phosphorylation, increased total YAP1 levels, and, consequently, elevated cotranscriptional activity of YAP1 in vivo. Most importantly, ablation of NDR in vivo deregulates YAP1 levels and activity without obviously altering MST1/2-LATS1/2 signaling, strongly suggesting that the observed deregulation of YAP1 is a direct effect of the absence of NDR kinases.

Mammalian NDR kinases are the closest homologs of LATS kinases [12], the only established YAP1-S127 kinases so far. NDR and LATS kinases efficiently phosphorylate the same synthetic substrate peptide [39, 40] and a peptide based on the sequence surrounding S127 on YAP1 [32]. Moreover, NDR and LATS kinases appear to be regulated in a similar fashion [3]. MST1/2 kinases can function as upstream kinases of LATS [41] and NDR [14, 17], and hMOB1 proteins act as coactivators for both NDR [14, 17, 42–44] and LATS kinases [39, 45–47]. Similar regulatory parallels have been observed in flies, namely that Hippo, the fly homolog of mammalian MST1/2, functions upstream of both Lats and Trc, the fly counterparts of NDR/LATS [48]. Likewise, the coactivator Mats/dMOB1, the homolog of hMOB1, regulates Lats and Trc in flies [49, 50]. Therefore, given that NDR and LATS kinases share similar regulatory mechanisms and substrate signatures [3], our discovery of NDR as a novel S127 kinase fits perfectly into the context of previously published data. Furthermore, our findings, together with the published regulatory similarities, suggest that MST1/2-MOB1 signaling might use diverse routes to regulate YAP1 phosphorylation. Therefore, future studies of NDR/Trc signaling downstream of MST/Hippo and MOB1/Mats are warranted in yet-to-be-established animal models.

Mechanistically, NDR-mediated YAP1-S127 phosphorylation drives cytoplasmic sequestration of YAP1 and suppresses YAP1-driven reporter activity (Figure 6). These observations recapitulate the effects reported for LATS on YAP1 in tissue-cultured cells [4]. Functionally and in full support of our finding that NDR negatively regulates YAP1 activity, NDR impairs proliferation and colony formation of YAP1-dependent colon cancer cells (Figure 6). Conversely, combined loss of murine

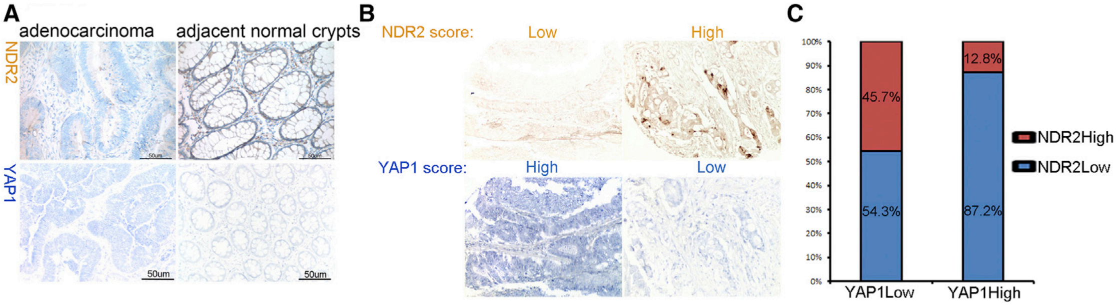

Figure 7. NDR2 and YAP1 Levels Are Inversely Correlated in Human Colon Cancer Samples

(A) YAP1 (blue) and NDR2 (brown) IHC staining in human colon adenocarcinoma and adjacent normal colon crypts. Scale bars represent 50 μm.

(B) Representative images of YAP1 and NDR2 IHC staining score in human colon cancer tissue microarrays (n = 400).

(C) NDR2 expression in tissue microarray samples with high versus low YAP1 levels. Absolute sample numbers are provided in the Supplemental Experimental Procedures.

See also Table S3.

NDR1/2 in the intestinal epithelium results in reduced YAP1 phosphorylation, whereas total YAP1 protein levels are increased (Figure 4). On a functional level in the intestine, YAP1 transcriptional coactivator activity is increased (Figure 4) and the proliferative zone of the epithelium is extended (Figure 1). Therefore, to our knowledge, the *Ndr* KO mice described in this study are the first animal model providing comprehensive in vivo evidence of a direct YAP1-S127 kinase. Follow-up studies are now needed to examine whether NDRs also function as S127 kinases in other organs such as the liver, whose tissue homeostasis is tightly regulated by YAP1 phosphorylation [9].

Although numerous studies have investigated S127 phosphorylation and its impact on YAP1 regulation (reviewed in [1, 30]), additional sites on YAP1 are phosphorylated by LATS, namely S61, S109, S164, and S381 [4, 32]. Phosphorylation at S381 primes YAP1 for proteasome-mediated degradation [31], whereas the function of the other phospho sites has remained enigmatic [3]. We found that NDR targets S61, S109, and S164 (Table S2) but did not observe S381 phosphorylation. In this context, the levels of phospho-S382, the murine equivalent of hYAP1 S381, are comparable in the intestinal epithelium of *N1/2* cDKO and control mice (Figure 4), and overexpression of active NDR does not increase S381 phosphorylation in tissue-cultured cells (Figure 5). Interestingly, total YAP1 protein levels are still increased in the absence of NDR (Figure 4) and decreased upon NDR overexpression (Figure 5), suggesting that alternative mechanisms to S381 phosphorylation must exist to regulate YAP1 protein levels in vivo. Whether these mechanisms are mediated by the other identified phosphorylation sites—S61, S109, or S164—or triggered by more indirect effects remains to be addressed with regard to NDR signaling.

Ablation of mammalian Hippo—*Mst1/2*—in the intestinal epithelium results in hyperproliferation through YAP1 upregulation [10]. However, the direct physiological S127 kinase in this tissue was not defined experimentally [10]. Mice with specific LATS1/2 deletion in the intestinal epithelium have not been reported thus far. Instead, we show that NDR1/2 loss in the intestinal epithelium leads to increased proliferation and the formation of hyperplastic foci (Figure 1). Therefore, we are tempted to speculate that MST1/2 kinases restrict proliferation in the intestinal epithelium at least in part via NDR kinase signaling. However, loss of NDR1/2 in the intestinal epithelium results in a substantially weaker YAP1-dependent phenotype than ablation of *Mst1/2* [10] or *Sav1*, a scaffold protein of the MST1/2 kinase complex [11]. This indicates that MST1/2 kinases presumably regulate additional downstream targets distinct from NDR that impact on YAP1 regulation in the intestinal epithelium. Clearly more work is required to test whether LATS or other yet-to-be-identified YAP1 kinases/regulators play a role in controlling YAP1 in the intestinal epithelium.

Already-reported mouse models provide strong evidence suggesting that loss of S127 phosphorylation mimicked by the introduction of an S127A YAP1 mutant or loss of the MST1/2 upstream kinases is sufficient to drive hyperproliferation, expansion of progenitor cell compartments, and tumorigenesis in the intestinal epithelium [5, 10]. Although *N1/2* cDKO animals do not develop spontaneous tumors, they are exquisitely more sensitive to chemically induced colon carcinogenesis than wild-type controls (Figure 2). Significantly, this hypersensitivity is reduced by concomitant removal of one *Yap1* allele (Figure 4E), indicating that loss of NDR drives carcinogenesis through YAP1 in the intestinal epithelium. Importantly, we observe that only combined loss of both NDR1 and NDR2 gives rise to the full tumor phenotype (Figure 2) and hyperproliferation (Figure 1), supporting our earlier speculations [16] that NDR2 can partially compensate for the absence of NDR1 in *Ndr1* KO mice. In further support of a tumor-suppressive function of NDR2 in the intestinal epithelium, we found that loss of NDR2 correlates with tumor onset in wild-type mice with chemically induced carcinogenesis (Figure 3). Of equal importance, the majority of patient samples on a colon cancer tissue microarray displayed an inverse correlation between high YAP1 and low NDR2 levels (Figure 7). Collectively, these findings suggest that NDR2 may serve a tumor-suppressive role in human colorectal cancer.

In summary, we establish mammalian NDR as bona fide kinases phosphorylating YAP1 on the key regulatory site S127 in vitro and in vivo. NDR kinases function as tumor suppressors in the intestinal epithelium by negatively regulating YAP1. In general, our data strongly suggest that the contribution of NDR kinases to YAP1 regulation should be accounted for in future YAP1-related studies and reconsidered in settings where the nature of the direct S127 kinase has remained elusive. Collectively, our findings provide significant new insights for a broad range of research efforts aimed at decoding and eventually manipulating YAP1-driven biology with the aim of improving cancer treatment and regenerative medicine.

Experimental Procedures

Animal Experiments

All animal experiments were carried out in compliance with animal welfare regulation and approved by the Swiss Cantonal Veterinary Office of Basel.

Colon Carcinogenesis Model

Adult mice (aged 10 weeks, body weight $\geq$20 g) of the indicated *Ndr* genotypes were injected intraperitoneally with 7.4 mg/kg body weight AOM (Sigma) on day 1. From day 14 to day 21, drinking water was supplemented with 2% DSS (MP Biomedicals). On day 64, mice were sacrificed and tissue samples were collected for analysis.

Human Colon Cancer Tissue Microarray

A tissue microarray (TMA) of unselected, nonconsecutive human colorectal cancer (CRC) samples was described previously [51]. In brief, formalin-fixed, paraffin-embedded tissue blocks of CRC resections were retrieved from the archives of the Institute of Pathology, University Hospital Basel, and the Institute of Clinical Pathology, Basel. A table with clinicopathological features of each sample is available upon request. Failure of analysis (<10% of all cases) was related to TMA technology, including missing samples or fractions containing only a few tumor cells. The NDR2 and YAP IHC staining protocols were identical to those established for mouse samples (see the Supplemental Experimental Procedures).

See the Supplemental Experimental Procedures for detailed descriptions of kinase assays, tissue-culture experiments, plasmids, antibodies, and IHC.

Supplemental Information

Supplemental Information includes Supplemental Experimental Procedures, four figures, and three tables and can be found with this article online at http://dx.doi.org/10.1016/j.cub.2014.11.054.

Acknowledgments

We thank J.-F. Spetz and his team at the Friedrich Miescher Institute for Biomedical Research (FMI) for their mouse work assistance. M. Kirschmann helped with image quantification. A. Bogucki performed histological staining. F.T. and D.S-R. received support from the Swiss National Science Foundation (31003A_138287). A.H. is a Wellcome Trust Research Career Development fellow (090090/Z/09/Z). The FMI is supported by the Novartis Research Foundation.

Received: September 22, 2014
Revised: November 3, 2014
Accepted: November 20, 2014
Published: January 15, 2015

References

1. Hong, W., and Guan, K.L. (2012). The YAP and TAZ transcription co-activators: key downstream effectors of the mammalian Hippo pathway. Semin. Cell Dev. Biol. *23*, 785–793.
2. Harvey, K., and Tapon, N. (2007). The Salvador-Warts-Hippo pathway — an emerging tumour-suppressor network. Nat. Rev. Cancer *7*, 182–191.
3. Hergovich, A. (2013). Regulation and functions of mammalian LATS/NDR kinases: looking beyond canonical Hippo signalling. Cell Biosci. *3*, 32.
4. Zhao, B., Wei, X., Li, W., Udan, R.S., Yang, Q., Kim, J., Xie, J., Ikenoue, T., Yu, J., Li, L., et al. (2007). Inactivation of YAP oncoprotein by the Hippo pathway is involved in cell contact inhibition and tissue growth control. Genes Dev. *21*, 2747–2761.
5. Camargo, F.D., Gokhale, S., Johnnidis, J.B., Fu, D., Bell, G.W., Jaenisch, R., and Brummelkamp, T.R. (2007). YAP1 increases organ size and expands undifferentiated progenitor cells. Curr. Biol. *17*, 2054–2060.
6. Dong, J., Feldmann, G., Huang, J., Wu, S., Zhang, N., Comerford, S.A., Gayyed, M.F., Anders, R.A., Maitra, A., and Pan, D. (2007). Elucidation of a universal size-control mechanism in *Drosophila* and mammals. Cell *130*, 1120–1133.
7. Lu, L., Li, Y., Kim, S.M., Bossuyt, W., Liu, P., Qiu, Q., Wang, Y., Halder, G., Finegold, M.J., Lee, J.S., and Johnson, R.L. (2010). Hippo signaling is a potent in vivo growth and tumor suppressor pathway in the mammalian liver. Proc. Natl. Acad. Sci. USA *107*, 1437–1442.
8. Song, H., Mak, K.K., Topol, L., Yun, K., Hu, J., Garrett, L., Chen, Y., Park, O., Chang, J., Simpson, R.M., et al. (2010). Mammalian Mst1 and Mst2 kinases play essential roles in organ size control and tumor suppression. Proc. Natl. Acad. Sci. USA *107*, 1431–1436.
9. Zhou, D., Conrad, C., Xia, F., Park, J.S., Payer, B., Yin, Y., Lauwers, G.Y., Thasler, W., Lee, J.T., Avruch, J., and Bardeesy, N. (2009). Mst1 and Mst2 maintain hepatocyte quiescence and suppress hepatocellular carcinoma development through inactivation of the Yap1 oncogene. Cancer Cell *16*, 425–438.
10. Zhou, D., Zhang, Y., Wu, H., Barry, E., Yin, Y., Lawrence, E., Dawson, D., Willis, J.E., Markowitz, S.D., Camargo, F.D., and Avruch, J. (2011). Mst1 and Mst2 protein kinases restrain intestinal stem cell proliferation and colonic tumorigenesis by inhibition of Yes-associated protein (Yap) overabundance. Proc. Natl. Acad. Sci. USA *108*, E1312–E1320.
11. Cai, J., Zhang, N., Zheng, Y., de Wilde, R.F., Maitra, A., and Pan, D. (2010). The Hippo signaling pathway restricts the oncogenic potential of an intestinal regeneration program. Genes Dev. *24*, 2383–2388.
12. Hergovich, A., Stegert, M.R., Schmitz, D., and Hemmings, B.A. (2006). NDR kinases regulate essential cell processes from yeast to humans. Nat. Rev. Mol. Cell Biol. *7*, 253–264.
13. Stegert, M.R., Tamaskovic, R., Bichsel, S.J., Hergovich, A., and Hemmings, B.A. (2004). Regulation of NDR2 protein kinase by multi-site phosphorylation and the S100B calcium-binding protein. J. Biol. Chem. *279*, 23806–23812.
14. Hergovich, A., Kohler, R.S., Schmitz, D., Vichalkovski, A., Cornils, H., and Hemmings, B.A. (2009). The MST1 and hMOB1 tumor suppressors control human centrosome duplication by regulating NDR kinase phosphorylation. Curr. Biol. *19*, 1692–1702.
15. Hergovich, A., Lamla, S., Nigg, E.A., and Hemmings, B.A. (2007). Centrosome-associated NDR kinase regulates centrosome duplication. Mol. Cell *25*, 625–634.
16. Cornils, H., Stegert, M.R., Hergovich, A., Hynx, D., Schmitz, D., Dirnhofer, S., and Hemmings, B.A. (2010). Ablation of the kinase NDR1 predisposes mice to the development of T cell lymphoma. Sci. Signal. *3*, ra47.
17. Vichalkovski, A., Gresko, E., Cornils, H., Hergovich, A., Schmitz, D., and Hemmings, B.A. (2008). NDR kinase is activated by RASSF1A/MST1 in response to Fas receptor stimulation and promotes apoptosis. Curr. Biol. *18*, 1889–1895.
18. Cornils, H., Kohler, R.S., Hergovich, A., and Hemmings, B.A. (2011). Human NDR kinases control G(1)/S cell cycle transition by directly regulating p21 stability. Mol. Cell. Biol. *31*, 1382–1395.
19. Chakraborty, A., Prasanth, K.V., and Prasanth, S.G. (2014). Dynamic phosphorylation of HP1α regulates mitotic progression in human cells. Nat. Commun. *5*, 3445.
20. Chiba, S., Ikeda, M., Katsunuma, K., Ohashi, K., and Mizuno, K. (2009). MST2- and Furry-mediated activation of NDR1 kinase is critical for precise alignment of mitotic chromosomes. Curr. Biol. *19*, 675–681.
21. Madison, B.B., Dunbar, L., Qiao, X.T., Braunstein, K., Braunstein, E., and Gumucio, D.L. (2002). *cis* elements of the villin gene control expression in restricted domains of the vertical (crypt) and horizontal (duodenum, cecum) axes of the intestine. J. Biol. Chem. *277*, 33275–33283.
22. Yamada, Y., and Mori, H. (2003). Pre-cancerous lesions for colorectal cancers in rodents: a new concept. Carcinogenesis *24*, 1015–1019.
23. Whitehead, R.H., Demmler, K., Rockman, S.P., and Watson, N.K. (1999). Clonogenic growth of epithelial cells from normal colonic mucosa from both mice and humans. Gastroenterology *117*, 858–865.
24. Hill, M.J. (1983). Bile, bacteria and bowel cancer. Gut *24*, 871–875.
25. De Robertis, M., Massi, E., Poeta, M.L., Carotti, S., Morini, S., Cecchetelli, L., Signori, E., and Fazio, V.M. (2011). The AOM/DSS murine model for the study of colon carcinogenesis: from pathways to diagnosis and therapy studies. J. Carcinog. *10*, 9.
26. Maltzman, T., Whittington, J., Driggers, L., Stephens, J., and Ahnen, D. (1997). AOM-induced mouse colon tumors do not express full-length APC protein. Carcinogenesis *18*, 2435–2439.
27. Takahashi, M., Nakatsugi, S., Sugimura, T., and Wakabayashi, K. (2000). Frequent mutations of the β-catenin gene in mouse colon tumors induced by azoxymethane. Carcinogenesis *21*, 1117–1120.
28. Oving, I.M., and Clevers, H.C. (2002). Molecular causes of colon cancer. Eur. J. Clin. Invest. *32*, 448–457.
29. Halder, G., and Johnson, R.L. (2011). Hippo signaling: growth control and beyond. Development *138*, 9–22.
30. Harvey, K.F., Zhang, X., and Thomas, D.M. (2013). The Hippo pathway and human cancer. Nat. Rev. Cancer *13*, 246–257.

31. Zhao, B., Li, L., Tumaneng, K., Wang, C.Y., and Guan, K.L. (2010). A coordinated phosphorylation by Lats and CK1 regulates YAP stability through SCF(β-TRCP). Genes Dev. *24*, 72–85.
32. Hao, Y., Chun, A., Cheung, K., Rashidi, B., and Yang, X. (2008). Tumor suppressor LATS1 is a negative regulator of oncogene YAP. J. Biol. Chem. *283*, 5496–5509.
33. Tumaneng, K., Schlegelmilch, K., Russell, R.C., Yimlamai, D., Basnet, H., Mahadevan, N., Fitamant, J., Bardeesy, N., Camargo, F.D., and Guan, K.L. (2012). YAP mediates crosstalk between the Hippo and PI(3)K–TOR pathways by suppressing PTEN via miR-29. Nat. Cell Biol. *14*, 1322–1329.
34. Radu, A., Neubauer, V., Akagi, T., Hanafusa, H., and Georgescu, M.M. (2003). PTEN induces cell cycle arrest by decreasing the level and nuclear localization of cyclin D1. Mol. Cell. Biol. *23*, 6139–6149.
35. Zhao, B., Ye, X., Yu, J., Li, L., Li, W., Li, S., Yu, J., Lin, J.D., Wang, C.Y., Chinnaiyan, A.M., et al. (2008). TEAD mediates YAP-dependent gene induction and growth control. Genes Dev. *22*, 1962–1971.
36. Cook, D., Hoa, L.Y., Gomez, V., Gomez, M., and Hergovich, A. (2014). Constitutively active NDR1-PIF kinase functions independent of MST1 and hMOB1 signalling. Cell. Signal. *26*, 1657–1667.
37. Dupont, S., Morsut, L., Aragona, M., Enzo, E., Giulitti, S., Cordenonsi, M., Zanconato, F., Le Digabel, J., Forcato, M., Bicciato, S., et al. (2011). Role of YAP/TAZ in mechanotransduction. Nature *474*, 179–183.
38. Avruch, J., Zhou, D., and Bardeesy, N. (2012). YAP oncogene overexpression supercharges colon cancer proliferation. Cell Cycle *11*, 1090–1096.
39. Hergovich, A., Schmitz, D., and Hemmings, B.A. (2006). The human tumour suppressor LATS1 is activated by human MOB1 at the membrane. Biochem. Biophys. Res. Commun. *345*, 50–58.
40. Millward, T.A., Heizmann, C.W., Schäfer, B.W., and Hemmings, B.A. (1998). Calcium regulation of Ndr protein kinase mediated by S100 calcium-binding proteins. EMBO J. *17*, 5913–5922.
41. Chan, E.H., Nousiainen, M., Chalamalasetty, R.B., Schäfer, A., Nigg, E.A., and Silljé, H.H. (2005). The Ste20-like kinase Mst2 activates the human large tumor suppressor kinase Lats1. Oncogene *24*, 2076–2086.
42. Bichsel, S.J., Tamaskovic, R., Stegert, M.R., and Hemmings, B.A. (2004). Mechanism of activation of NDR (nuclear Dbf2-related) protein kinase by the hMOB1 protein. J. Biol. Chem. *279*, 35228–35235.
43. Devroe, E., Erdjument-Bromage, H., Tempst, P., and Silver, P.A. (2004). Human Mob proteins regulate the NDR1 and NDR2 serine-threonine kinases. J. Biol. Chem. *279*, 24444–24451.
44. Hergovich, A., Bichsel, S.J., and Hemmings, B.A. (2005). Human NDR kinases are rapidly activated by MOB proteins through recruitment to the plasma membrane and phosphorylation. Mol. Cell. Biol. *25*, 8259–8272.
45. Bothos, J., Tuttle, R.L., Ottey, M., Luca, F.C., and Halazonetis, T.D. (2005). Human LATS1 is a mitotic exit network kinase. Cancer Res. *65*, 6568–6575.
46. Kim, M., Kim, M., Lee, S., Kuninaka, S., Saya, H., Lee, H., Lee, S., and Lim, D.S. (2013). cAMP/PKA signalling reinforces the LATS-YAP pathway to fully suppress YAP in response to actin cytoskeletal changes. EMBO J. *32*, 1543–1555.
47. Yabuta, N., Okada, N., Ito, A., Hosomi, T., Nishihara, S., Sasayama, Y., Fujimori, A., Okuzaki, D., Zhao, H., Ikawa, M., et al. (2007). Lats2 is an essential mitotic regulator required for the coordination of cell division. J. Biol. Chem. *282*, 19259–19271.
48. Emoto, K., Parrish, J.Z., Jan, L.Y., and Jan, Y.N. (2006). The tumour suppressor Hippo acts with the NDR kinases in dendritic tiling and maintenance. Nature *443*, 210–213.
49. He, Y., Emoto, K., Fang, X., Ren, N., Tian, X., Jan, Y.N., and Adler, P.N. (2005). *Drosophila* Mob family proteins interact with the related Tricornered (Trc) and Warts (Wts) kinases. Mol. Biol. Cell *16*, 4139–4152.
50. Lai, Z.C., Wei, X., Shimizu, T., Ramos, E., Rohrbaugh, M., Nikolaidis, N., Ho, L.L., and Li, Y. (2005). Control of cell proliferation and apoptosis by Mob as tumor suppressor, Mats. Cell *120*, 675–685.
51. Tornillo, L., Lugli, A., Zlobec, I., Willi, N., Glatz, K., Lehmann, F., Spichtin, H.P., Maurer, R., Stoios, D., Sauter, G., and Terracciano, L. (2007). Prognostic value of cell cycle and apoptosis regulatory proteins in mismatch repair-proficient colorectal cancer: a tissue microarray-based approach. Am. J. Clin. Pathol. *127*, 114–123.

Current Biology, Volume *25*

Supplemental Information

NDR Functions as a Physiological YAP1 Kinase in the Intestinal Epithelium

Lei Zhang, Fengyuan Tang, Luigi Terracciano, Debby Hynx, Reto Kohler, Sandrine Bichet, Daniel Hess, Peter Cron, Brian A. Hemmings, Alexander Hergovich, and Debora Schmitz-Rohmer

Supplemental Information

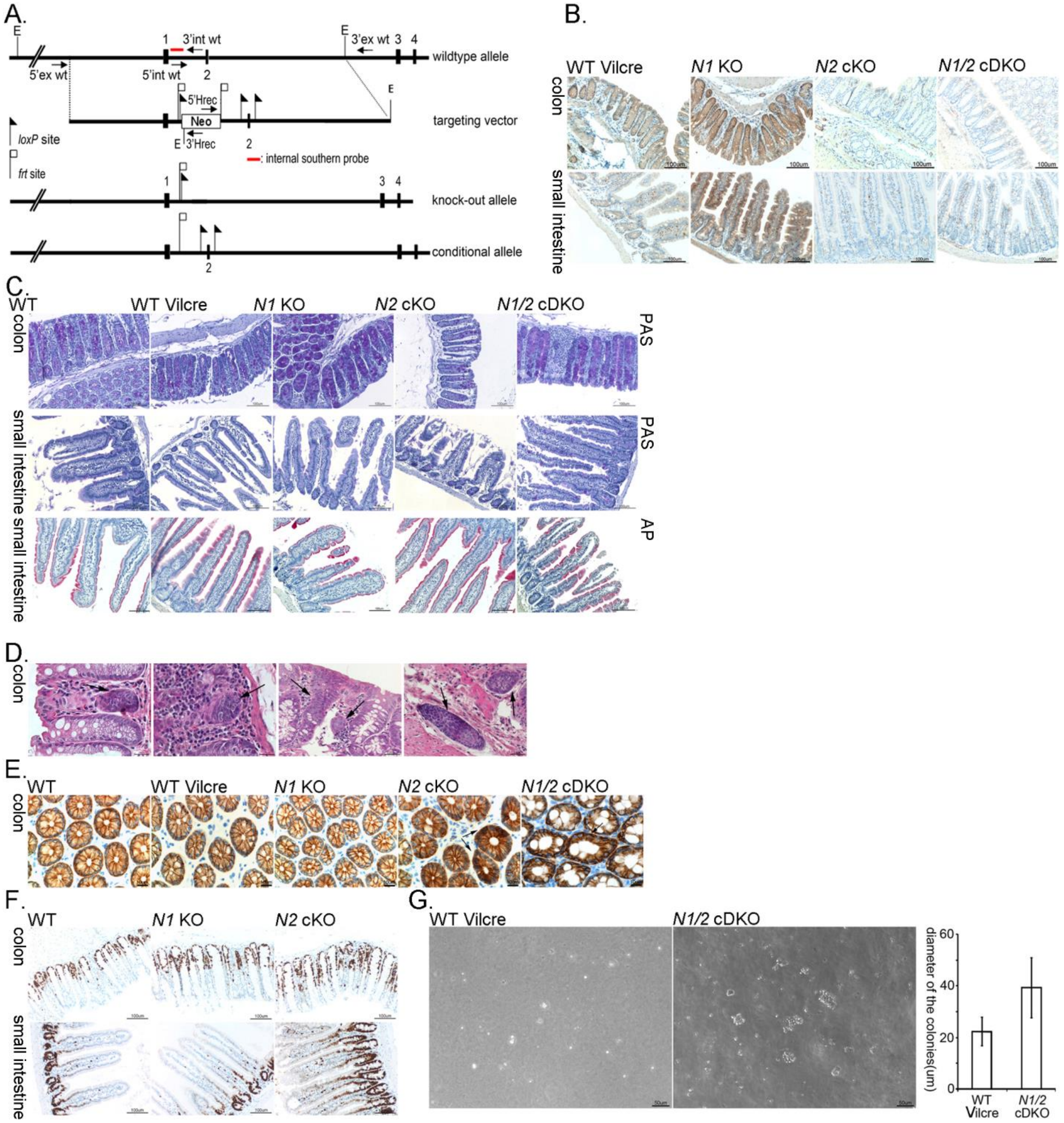

Figure S1 (related to Figure 1). Characterization of the *Ndr* knock-out in the intestinal epithelium

(A) Genomic structure of the *Ndr2* locus in the mouse and targeting vector for conditional *Ndr2* knock-out. Primer binding sites for ES cell screening are indicated (Ex com 5‘/3‘– common external 5‘/3‘ pimer; wt 5‘/3‘ – wild type internal primers; H rec 5‘/3‘ – homologous recombination primers in Neo cassette). E: EcoRI restriction sites used for Southern blot validation of single integration. Binding site of internal southern probe indicated in red. *frt* sites (empty squares) for removal of Neo cassette and *lox*P sites (solid triangles) for removal of Exon 2 are indicated. Note: The generation of the conditional *Ndr2* knock-out mouse line was part of a PhD thesis which can be accessed at http://edoc.unibas.ch/1455/1/Gebundene_Version.pdf.

(B) Validation of intestinal epithelial *Ndr2* knock-out model. NDR2 protein is absent from the intestinal epithelium of *N2* cKO and *N1/2* cDKO mice. Top: colon; bottom: small intestine

(C) Secretory lineage and absorptive enterocytes in *Ndr* single- and double- KO mice are indistinguishable from control mice. Periodic acid Schiff (PAS, top row: colon, middle row: small intestine) and Alkaline Phosphatase (AP, bottom row, small intestine) stainings. Genotypes are indicated above.

(D) Four representative examples of abnormal crypts (indicated by arrows) in *N1/2* cDKO mice present enlarged nuclei, thickened epithelial layers and loss of apical / basal polarity in a subset of cells.

(E) Beta-catenin accumulated crypts (BCAC) in *N2* cKO and *N1/2* cDKO colons, indicated by arrows. Beta-catenin levels in normal crypts are comparable between knock-out and control tissues. Genotypes are indicated above.

(F) Ki67 staining in wild-type and *Ndr* single-knock out intestines. Top: colon; bottom: small intestine. See Fig 1C, D for quantification.

(G) Colony formation assay with primary intestinal epithelial cells freshly isolated from control (WT VilCre) and *N1/2* cDKO colons. Cells from each genotype form similar numbers of colonies (data not shown) but the diameter of *N1/2* cDKO colonies is significantly bigger. $n = 50$; $p = 7.4 * 10^{-8}$

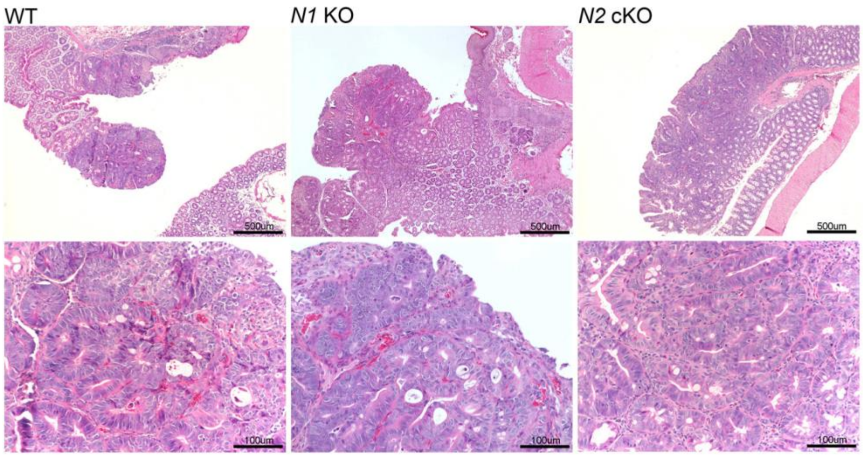

Figure S2 (related to Figure 2). Wild-type and *Ndr* single knock-out mice develop adenocarcinoma upon AOM/DSS treatment.

H&E stained sections of colon nodules in AOM/DSS-treated mice of indicated genotypes after dissection at d64 (see main Figure 2A for treatment protocol). Two different magnifications are shown.

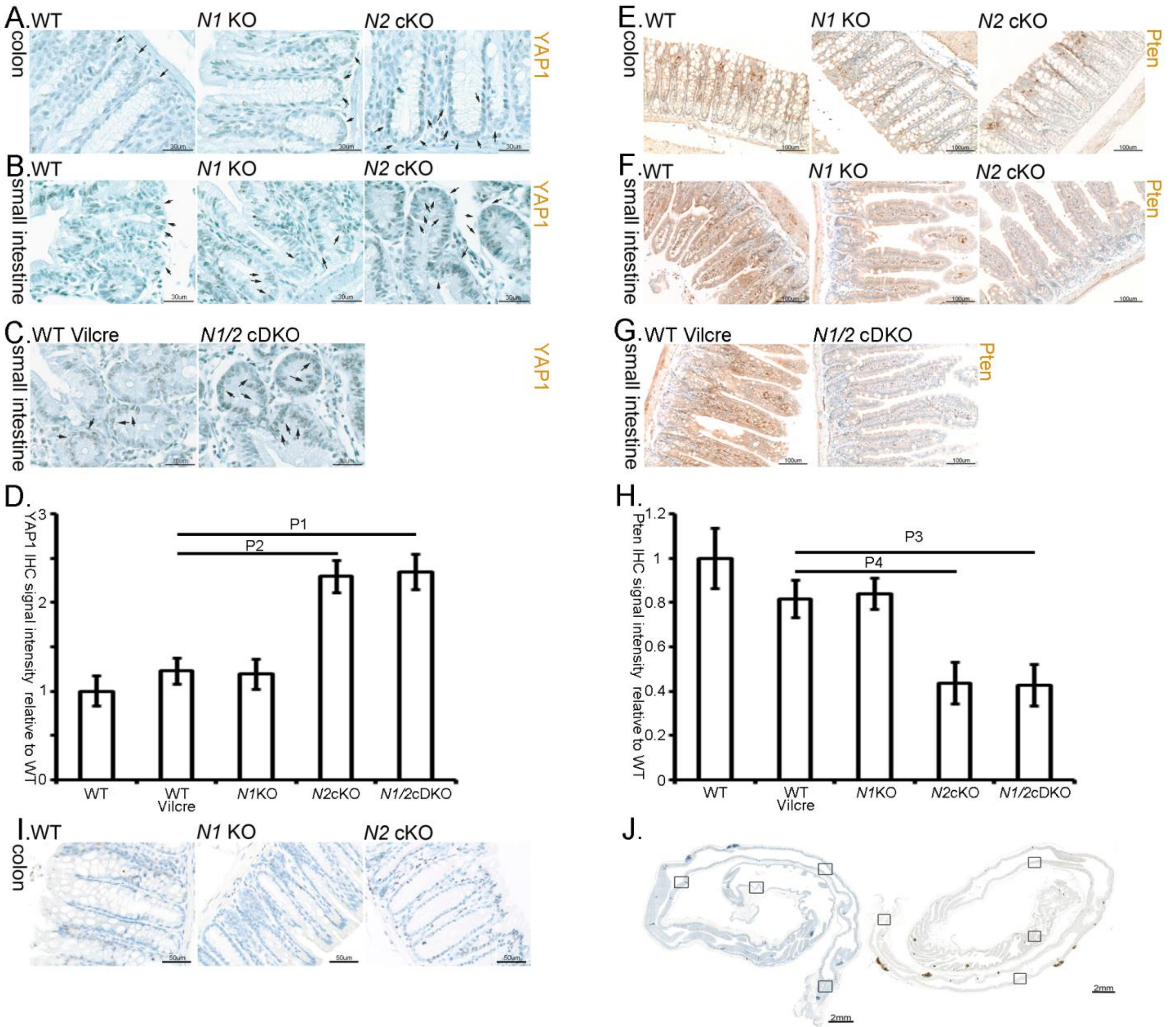

Figure S3 (related to Figure 4). YAP1 and PTEN IHC staining in *Ndr* knock-out mice.

(A), (B), (C) YAP1 protein (brown) detected by IHC in the intestinal epithelium. Genotypes are indicated above, tissue origin on the left.

(D) Quantification of YAP1 IHC signal in the colon (Fig 4B (WT Cre, *N1/2* cDKO) and S3A (WT, *N1* KO, *N2* cKO)). Signal in WT was set as 1. Details of the quantification

method are provided in the Supplemental Experimental Procedures section. T-test: p1 = $2 * 10^{-3}$; p2 = $2 * 10^{-3}$

(E), (F), (G) Pten protein (brown) detected by IHC in the intestinal epithelium. Genotypes are indicated above, tissue origin on the left.

(H) Quantification of Pten IHC signal in the intestinal epithelium (Fig 4C (WT Cre, *N1/2* cDKO) and S3E - G (WT, *N1* KO, *N2* cKO)). Signal in WT was set as 1. Details of the quantification method are provided in the Supplemental Experimental Procedures section. T-test: p3 = $3 * 10^{-3}$; p4 = $3 * 10^{-3}$

(I) *Bdnf in situ* hybridization of wild-type and *Ndr* single knock-out colons.

(J) Squares indicate areas taken for quantification of *Bdnf* signal in control (WT VilCre) and *N1/2* cDKO colons – quantification results shown in Fig 4D. Details of the quantification method are provided in the Supplemental Experimental Procedures section.

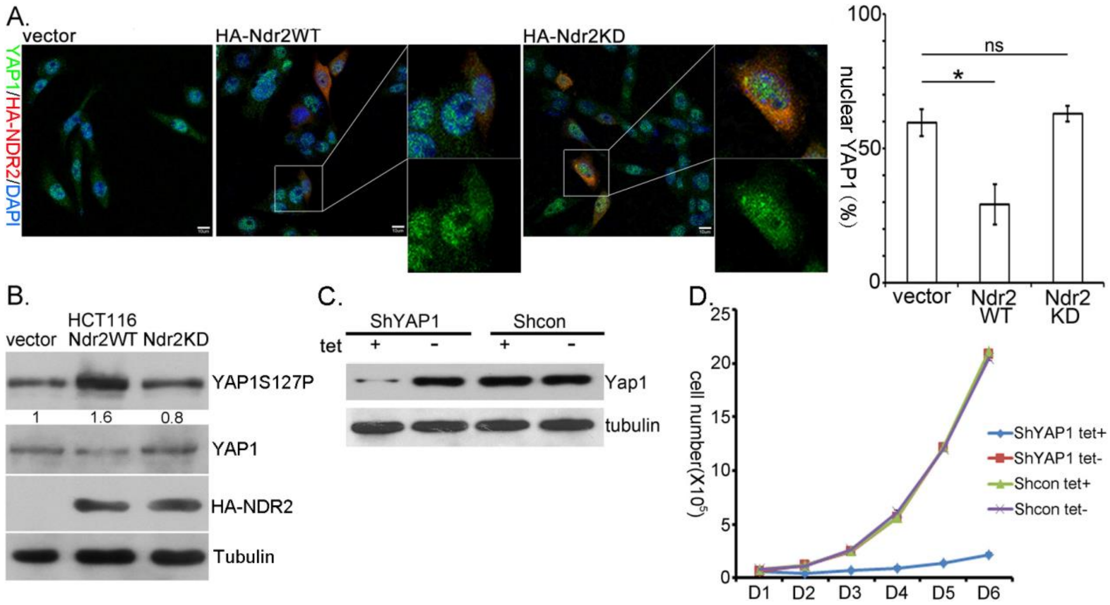

Figure S4 (related to Figure 6). Transient overexpression of active NDR changes YAP regulation

(A) Transient overexpression of wild-type (WT) but not kinase-dead (KD) HA-Ndr2 reduces nuclear localization of endogenous YAP1 in SW480 cells. Quantification on the right; n = 50 cells per condition. p = 0.002.

(B) Overexpression of wild-type (WT) but not kinase-dead (KD) Ndr2 increases S127 phosphorylation of endogenous Yap1 in HCT116 cells.

(C) Validation of tet-induced YAP1 knock-down in SW480 cells.

(D) Tet-induced knock-down of YAP1 reduces proliferation of SW480 cells as reported previously [S1].

Table S1

Mouse ID	*Ndr1*	*Ndr2*	nodule number
1	-/-	Δ/Δ	13
2	-/-	Δ/Δ	18
3	-/-	Δ/Δ	17
4	-/-	Δ/Δ	16
5	-/-	Δ/Δ	15
6	+/+	+/+	2
7	+/+	+/+	0
8	+/+	+/+	2
9	+/+	+/+	6
10	+/+	+/+	1
11	+/+	Δ/Δ	6
12	+/+	Δ/Δ	7
13	+/+	Δ/Δ	5
14	-/-	+/+	3
15	-/-	+/+	1
16	-/-	+/+	2

Number of colonic nodules formed in mice with indicated genotypes upon AOM/DSS treatment. Δ denotes conditional ablation of *Ndr2* in the intestinal epithelium. All mice were female and carried the Vilin-cre transgene.

Table S2

p-site	Peptide Sequence	Mascot Score
S109	QAsTDAGTAGALTPQHVR	60.07
S61 or T63	GD[set]DLEALFNAVMNPK	102.6
S127 or S128 or S131	AH[ssPAs]LQLGAVSPGTLTPT GVVSGPAATPTAQHLR	46
S163 or S164	QsSFEIPDDVPLPAGWEMAK	46.68
	QSsFEIPDDVPLPAGWEMAK	48.89

Identification of additional sites on YAP1 that are phosphorylated by recombinant NDR.

YAP1 was subjected to *in vitro* phosphorylation by NDR, and subsequently purified by SDS-PAGE. Yap1 bands were excised from the gel, subjected to tryptic digest and analyzed by NanoLC-MS/MS on a LTQ Orbitrap Velos (Thermo Scientific) as described [S2]. The phosphorylated peptides were identified with Mascot searching Swiss-Prot 2013_11 [S3] and validated with ScaffoldPTM (Proteome Software).

The sequences of the identified phosphopeptides are shown and the phosphorylated amino acids are marked in red. In cases where the phosphorylation could not be unambiguously assigned to a single serine or threonine in a peptide, the region of phosphorylation is marked in brackets and the possible phosphorylation sites are shown in lower case. All peptides were sequenced multiple times and the highest Mascot Score obtained for each peptide is shown.

Table S3

		YAP1 score	
		Low	High
NDR2 score	Low	51	197
	High	43	34

YAP1 and NDR2 protein expression score in tissue microarray samples. Data are shown as bar graphs in Figure 7C.

Supplemental Experimental Procedures

Histology

Animals were sacrificed, intestines removed, rinsed in cold PBS and fixed overnight in 10% formalin. Following dehydration, tissues were embedded in paraffin in a tissue processor (Medite, TCP15). 3 um sections were cut on a microtome (Microm HM355S) and stained with hematoxylin and eosin (H&E) or used for immunohistochemical stainings.

Alkaline phosphatase activity was assessed with the Vector Red Alkaline Phosphatase Substrate Kit I (Vectorlabs) according to the manufacturer's instructions and counterstained with hematoxylin. For periodic acid-Schiff (PAS) staining, sections were deparaffinized, immersed in Periodic Acid Solution (Sigma) for 5 min at room temperature, washed in water, and incubated in Schiff's reagent (Sigma) for 15 min at room temperature. Sections were washed and counterstained with hematoxylin.

Immunohistochemistry (IHC)

IHC stainings were performed on the Ventana DiscoveryXT instrument (Roche Diagnostics) with either Research IHC DAPMap XT (Ki67, β-catenin, Pten and Ndr2) or Research IHC BlueMap XT (YAP1) procedures. Briefly, for Ndr2 (1:50) and Pten (1:100) slides were pre-treated with standard CC1 (Roche Diagnostics), for β-catenin (1:400) slides were pre-treated with standard CC2 (Roche Diagnostics), for Ki67 (1:50) and Yap1 (1:200) slides were pre-treated with mild CC1 (Roche Diagnostics). Detection of primary antibodies was obtained by manually adding an anti-rabbit polymer conjugated

with HRP (ImmPress Vector) for Ndr2, Pten, β-catenin and Ki67, whereas detection of Yap1 primary antibody was obtained by adding a biotinylated Donkey anti-rabbit secondary antibody (Jackson Laboratories). In addition, for detection of β-catenin we added a rabbit anti-mouse monoclonal antibody (Epitomics) to reduce non-specific binding of the primary antibody. Finally, DAB Map kit (Roche Diagnostics) was used for visualization of antibodies binding to NDR2, Pten, β-catenin and Ki67 and Blue Map kit was used for YAP1.

Quantification of Ki67-positive cells (Fig 1C, D; S1D)

Five images per sample were collected at equal spacing at 10X magnification. Next, 50 crypts were randomly selected for each mouse and Ki67-positive cells were counted with the software Fiji [S4]. The data shown represent the average number of Ki67-positive cells in a given genotype based on five mice analyzed per genotype.

Quantification of YAP and Pten IHC (Fig 4B, C and S3A – H)

Five mice were analyzed per genotype. One section per mouse was photographed and images were segmented into different channels using the Ilastik program (Sommer, C; Straehle C; Koethe U; Hamprecht FA (2011). "ilastik: Interactive Learning and Segmentation Toolkit". *IEEE International Symposium on Biomedical Imaging*: 230–33.). Segmented images where further processed with Fiji [S4] to obtain the percentage of total area covered by YAP or Pten IHC signal. Values obtained for each genotype were normalized to the WT value.

mRNA in situ hybridization with ACD probes and quantification

In Situ Hybridization (ISH) was performed on Ventana Discovery XT instrument using RNAscope technique with the following probes: BDNF (ACD # 316036) and CTGF (ACD # 314546) on 5 um thick colon and small intestine paraffin sections. Briefly, slides were heated to 60°C for 45min and dewaxed up to 100% EtOH offline and air dried. Next, sections were put into pre-heated pretreat2 buffer (ACD) and heated to 98°C for 8 minutes in a microMED T/T Mega (Milestone) microwave. Slides were rinsed in water and put into the instrument for automated staining. The procedure (mRNA DAB DXT 2.0) follows this setting: pre-treatment 2 for 4 min, pre-treatment 3 for 8 min, hybridization with the probes at 48°C for 2h 32 min, Amp5 for 24 min. DapB (ACD # 310048) was used as negative control probe and PPIB (ACD # 313916) was used as positive control probe. BDNF ISH slides were scanned with an Axioscan scanner (Zeiss) and images with an area of 1mm^2 from cecum (1 image), colon (2 images) and rectum (1 image) were taken for quantification as indicated in Fig S3J. Images were segmented into different channels using the Ilastik program ("ilastik: Interactive Learning and Segmentation Toolkit". *IEEE International Symposium on Biomedical Imaging*: 230–33.). Segmented images where further processed with Fiji [S4] to obtain the ratio of the total tissue area covered by BDNF signal.

Isolation and colony formation of murine intestinal epithelial cells (IEC)

IECs were isolated according to a protocol established by Whitehead et al. [S5]. Mice were sacrificed, colons were removed and placed in PBS. Contents of the colon were removed with PBS using a 10-ml syringe, then washed once with cold PBS containing

0.02% NaClO. Following transfer to a clean dish, colons were washed three times with fresh cold PBS to remove the NaClO. Colons were opened longitudinally and then cut into small pieces with a scalpel. The colonic epithelium was detached using forceful shaking in 0.5 mM EDTA and 0.5 mM dithiothreitol (DTT) in PBS for 30 minutes at 4°C. Muscle tissue fragments were allowed to settle down by gravity for 2 minutes. The supernatant containing the IECs was collected and treated with collagenase (75 ug / ml), protease (20 ug / ul) and Dnase (20 ug /ml) for 15 Minutes at 37°C to generate a single-cell suspension. Next, cells were washed three times with fresh PBS to remove all enzymes. Cells were seeded into agarose plates containing 0.1% agarose in RPMI1640 with 10% FBS. Cells were cultured at 37°C, 5% CO_2, for 10 days to form colonies. An LSM700 confocal system was used to acquire pictures for quantification. Ten images were collected randomly at equal spacing at X20 magnification and colonies were measured and quantified by the IMS image processing software.

Plasmids and antibodies

Antibodies used in the study were obtained from the following suppliers: anti-Ki67 antibody (RM-9106) – Thermo Scientific; anti-YAP1 (#4912), anti-YAP1S127P (#4911), anti-Pten (#9559), anti-LATS1 (# 9153), anti-LATS1S909P (#9157), anti-LATS1T1079P (#8654), anti-MST1 (#3682), anti-MST1/2(T180/T183)P (#3681) – Cell Signaling Technology; anti-β-catenin (14/Beta-Catenin) – BD Transduction Laboratories; anti-YAP1 (sc-15407) for immuno-histochemistry (IHC) – Santa Cruz Biotechnology. Anti-HA Tag antibody (12CA5) – home-made. Rabbit polyclonal anti-NDR1, anti-NDR and anti-p444/2 were generated by us and have been published previously ([S6, S7]).

pcDNA3.0-HA-Ndr1 (wild-type), pcDNA3.0-HA-Ndr1 (kinase dead), pcDNA3.0-HA-Ndr2 (wildtype), pcDNA3.0-HA-Ndr2 (kinase dead) for expression in mammalian cells have been described [S8]. GST-tagged NDR1 PIF (wild-type and kinase-dead, [S9]) were cloned into pFastbac1 and expressed in Sf9 insect cells. pGEX3X-hYAP1 was used for bacterial expression of GST-tagged YAP1. The TEAD-luciferase reporter plasmid was obtained from Addgene (Plasmid 34615; 8xGTIIC-luciferase). pGIPZ-Sh Yap1 and pGIPZ-Sh Luc plasmids were a kind gift from Zhou Dawang.

NDR kinase assays

NDR kinase assays were performed as described in [S8]. Briefly, kinase reactions were carried out in 30 µl buffer containing 20 mM Tris, pH 7.5, 10 mM $MgCl_2$, 1 mM benzamidine, 4 µM leupeptin, 1 µM microcystin, 1 mM DTT, 1 µM cyclic AMP-dependent protein kinase inhibitor peptide, 1 µM ATP, 5 µCi P32 with 500 ng GST-NDR kinase and 500 ng GST-YAP1 protein. Reactions were incubated for 30 minutes at 37°C, stopped by addition of 10 ul 5x Lämmli buffer and boiled for 5 minutes at 95°C prior to resolving them on an SDS-PAGE gel. Proteins were transferred to an immobilon-P PVDF membrane (Millipore), exposed to a phospho-screen and read on a phospho-imager.

Cell culture and transfection

SW480 cells were bought from ATCC (CCL-228) and cultured in RPMI1640 medium with 10% FBS. For transfection, 5 *10^5 (6-well dish) or 2*10^6 (10 cm dish) were plated 24h prior to transfecting with lipofectamine 2000 according to the manufacturer's

instructions. Stable SW480 cell lines expressing Ndr2 wild-type, Ndr2 kinase-dead or luciferase upon tetracycline (tet) administration were generated under selection with Blasticidin (10 ug / ml) and G418 (1 mg / ml).

Immunofluorescence

SW480 cells were seeded in chamber-slides and transfected as described above. 24h after transfection, cells were fixed with 4%PFA for 10min on ice. Next, cells were washed once with PBS for 5 minutes, followed by incubation with 0.5% Triton X-100 in PBS for 15 minutes on ice. After two washes 5-minute washes with PBS, cells were blocked with 1% BSA in PBS at 37°C for 30 minutes. Next, cells were incubated over night at 4°C with primary antibody (anti-Yap1 (1/500) from CST (#4912) and anti-HA (12CA5, homemade, 1/200) in PBS containing 1%BSA. Slides were washed twice with PBS, 5 minutes each time. For detection, cells were incubated with Alexa 647 anti-rabbit (1/1000) and Alexa 488 anti-mouse (1/1000) in PBS for 1h at 37°C. After two 5 – minute washes with PBS, cells were incubated with 5ug / ml DAPI in PBS for 2 minutes at RT. Prolong@Gold medium (Invitrogen) was used to mount slides. An LSM700 confocal system was used to analyze slides. To determine the percentage of nuclear YAP (Fig 6A, S6A), 50 HA-positive cells were collected randomly at equal spacing at 40X magnification. YAP1 signal density for the nucleus (based on DAPI co-localization) and YAP signal density for the entire cell were measured with the IMS image processing software.

TEAD-reporter assay

SW480 cells were seeded in 24-well plates at 100000 cells / well 24h prior to triple-transfection with TEAD-Luciferase reporter (470 ng / well), renilla firefly (10 ng / well) and Ndr2WT (60 ng / well) or Ndr2KD (60 ng / well). 24 hours after transfection, cells were lysed and luciferase activity was assayed using the Dual-Luciferase® Reporter Assay System (Promega, E1910) following the manufacturer's instructions.

Proliferation assays

Stable tet-inducible SW480 cell lines were seeded into 6 well dishes at 5000 cells / well on day 0 in tet-containing medium. Medium was changed every 48h and fresh tet was added. At each time point, cells were detached with 0.5 mM EDTA in PBS and counted with a Vi-CELL analyser (Beckmann Coulter).

References

S1. Zhou, D., Zhang, Y., Wu, H., Barry, E., Yin, Y., Lawrence, E., Dawson, D., Willis, J.E., Markowitz, S.D., Camargo, F.D., et al. (2011). Mst1 and Mst2 protein kinases restrain intestinal stem cell proliferation and colonic tumorigenesis by inhibition of Yes-associated protein (Yap) overabundance. Proceedings of the National Academy of Sciences of the United States of America *108*, E1312-1320.

S2. Grzmil, M., Huber, R.M., Hess, D., Frank, S., Hynx, D., Moncayo, G., Klein, D., Merlo, A., and Hemmings, B.A. (2014). MNK1 pathway activity maintains protein synthesis in rapalog-treated gliomas. J Clin Invest *124*, 742-754.

S3. Perkins, D.N., Pappin, D.J., Creasy, D.M., and Cottrell, J.S. (1999). Probability-based protein identification by searching sequence databases using mass spectrometry data. Electrophoresis *20*, 3551-3567.

S4. Schindelin, J., Arganda-Carreras, I., Frise, E., Kaynig, V., Longair, M., Pietzsch, T., Preibisch, S., Rueden, C., Saalfeld, S., Schmid, B., et al. (2012). Fiji: an open-source platform for biological-image analysis. Nat Methods *9*, 676-682.

S5. Whitehead, R.H., Demmler, K., Rockman, S.P., and Watson, N.K. (1999). Clonogenic growth of epithelial cells from normal colonic mucosa from both mice and humans. Gastroenterology *117*, 858-865.

S6. Cornils, H., Stegert, M.R., Hergovich, A., Hynx, D., Schmitz, D., Dirnhofer, S., and Hemmings, B.A. (2010). Ablation of the kinase NDR1 predisposes mice to the development of T cell lymphoma. Science signaling *3*, ra47.
S7. Tamaskovic, R., Bichsel, S.J., Rogniaux, H., Stegert, M.R., and Hemmings, B.A. (2003). Mechanism of Ca2+-mediated regulation of NDR protein kinase through autophosphorylation and phosphorylation by an upstream kinase. J Biol Chem *278*, 6710-6718.
S8. Hergovich, A., Bichsel, S.J., and Hemmings, B.A. (2005). Human NDR kinases are rapidly activated by MOB proteins through recruitment to the plasma membrane and phosphorylation. Mol Cell Biol *25*, 8259-8272.
S9. Stegert, M.R., Tamaskovic, R., Bichsel, S.J., Hergovich, A., and Hemmings, B.A. (2004). Regulation of NDR2 protein kinase by multi-site phosphorylation and the S100B calcium-binding protein. J Biol Chem *279*, 23806-23812.
